# Functional Plasticity in the Type IV Secretion System of *Helicobacter pylori*

**DOI:** 10.1371/journal.ppat.1003189

**Published:** 2013-02-28

**Authors:** Roberto M. Barrozo, Cara L. Cooke, Lori M. Hansen, Anna M. Lam, Jennifer A. Gaddy, Elizabeth M. Johnson, Taryn A. Cariaga, Giovanni Suarez, Richard M. Peek, Timothy L. Cover, Jay V. Solnick

**Affiliations:** 1 Center for Comparative Medicine, University of California Davis, Davis, California, United States of America; 2 Department of Medicine, Vanderbilt University, School of Medicine, Nashville, Tennessee, United States of America; 3 Department of Microbiology and Immunology, Vanderbilt University, Nashville, Tennessee, United States of America; 4 Veterans Affairs Tennessee Valley Healthcare System, Nashville, Tennessee, United States of America; 5 Department of Medicine, University of California Davis, School of Medicine, Davis, California, United States of America; 6 Department of Microbiology and Immunology, University of California Davis, School of Medicine, Davis, California, United States of America; 7 California National Primate Research Center, University of California Davis, Davis School of Medicine, Davis, California, United States of America; Duke University, United States of America

## Abstract

*Helicobacter pylori* causes clinical disease primarily in those individuals infected with a strain that carries the cytotoxin associated gene pathogenicity island (*cag*PAI). The *cag*PAI encodes a type IV secretion system (T4SS) that injects the CagA oncoprotein into epithelial cells and is required for induction of the pro-inflammatory cytokine, interleukin-8 (IL-8). CagY is an essential component of the *H. pylori* T4SS that has an unusual sequence structure, in which an extraordinary number of direct DNA repeats is predicted to cause rearrangements that invariably yield in-frame insertions or deletions. Here we demonstrate in murine and non-human primate models that immune-driven host selection of rearrangements in CagY is sufficient to cause gain or loss of function in the *H. pylori* T4SS. We propose that CagY functions as a sort of molecular switch or perhaps a rheostat that alters the function of the T4SS and “tunes” the host inflammatory response so as to maximize persistent infection.

## Author Summary

*Helicobacter pylori* is a bacterium that colonizes the stomach of about half the world's population, most of whom are asymptomatic. However, some strains of *H. pylori* express a bacterial secretion system, a sort of molecular syringe that injects a bacterial protein inside the gastric cells and causes inflammation that can lead to peptic ulcer disease or gastric cancer. One of the essential components of the *H. pylori* secretion system is CagY, which is unusual because it contains a series of repetitive amino acid motifs that are encoded by a very large number of direct DNA repeats. Here we have shown that DNA recombination in *cagY* changes the protein motif structure and alters the function of the secretion system—turning it on or off. Using mouse and non-human primate models, we have demonstrated that CagY is a molecular switch that “tunes” the host inflammatory response, and likely contributes to persistent infection. Determining the mechanism by which CagY functions will enhance our understanding of the effects of *H. pylori* on human health, and could lead to novel applications for the modulation of host cell function.

## Introduction

*Helicobacter pylori* commonly infects the human gastric epithelium and sometimes causes peptic ulcer disease or gastric cancer, which is the second most common cause of cancer death worldwide. The *H. pylori* virulence locus most strongly associated with clinical disease, rather than asymptomatic infection, is the *cag* pathogenicity island (*cag*PAI). The 40-kb *cag*PAI consists of approximately 27 genes, several of which encode a type IV secretion system (T4SS) that binds β1 integrins [1], [2] and translocates the CagA oncoprotein into gastric epithelial cells [3]. Phosphorylated and nonphosphorylated forms of intracellular CagA cause complex changes in host-cell signaling that lead to epithelial cell elongation [4], disruption of tight junctions [5], and alteration of cell polarity [6], [7]. The T4SS is also required for induction of interleukin-8 (IL-8), a member of the CXC cytokine family, which has long been used as a convenient assay to characterize the inflammatory potential of *H. pylori* strains [8], [9]. It has been proposed that IL-8 induction is mediated by *cag*PAI-dependent translocation of peptidoglycan, activation of nucleotide-binding oligomerization domain 1 (NOD1), and stimulation of NF-κB [10]. However, this remains controversial, as some have suggested that IL-8 and other NF-κB-dependent proinflammatory responses are mediated primarily by toll like receptors and MyD88, rather than NOD1 [11]. Very recently, a NOD1- and CagA-independent pathway of IL-8 induction has also been described [12].

The prototypical T4SS is the VirB secretion apparatus of *Agrobacterium tumefaciens*, which consists of 11 VirB proteins (encoded by *virB1-11*) and the coupling protein, VirD4 [13]. Although the function of the *H. pylori* T4SS proteins cannot be easily assigned based on the distantly related *A. tumefaciens*, functional and structural studies are beginning to emerge. Mutagenesis studies have demonstrated that 15 genes on the *cag*PAI are required for *H. pylori* induction of IL-8 [14], [15]. One such gene is *cagY*, which encodes the *H. pylori* VirB10 orthologue. CagY is a large protein of approximately 220 kDa that is thought to mediate contact between the inner and outer bacterial membrane [16], similar to what has been described in *A. tumefaciens* and other Gram-negative bacteria [17]. However, *cagY* is much larger than *virB10* from *A. tumefaciens*, and it has an unusual sequence structure in which an extraordinary number of direct DNA repeats are found in a small 5′ repeat region (FRR) and a large middle repeat region (MRR) of the gene [18]. Potential DNA rearrangements predicted by these repeats invariably yield in-frame insertions or deletions that result in variant proteins. The observation that variant CagY proteins are found in different *H. pylori* strains or after passage in mouse models, led to the suggestion that CagY undergoes antigenic variation to evade the host immune response [18] while maintaining T4SS function [19].

Here we demonstrate that experimental infection with *H. pylori* leads to host immunity-dependent recombination in *cagY* that is sufficient to eliminate the functionality of the T4SS. Moreover, changes in *cagY* during experimental infection could also turn on the capacity to induce IL-8 and phosphorylate CagA, suggesting that the function of CagY diversity is not to evade the host immune response but rather to modulate it. We propose that CagY functions as a molecular switch or perhaps a rheostat that “tunes” the host inflammatory response by altering the function of the T4SS so as to maximize persistent infection.

## Results

### *H. pylori* isolates recovered from experimentally infected rhesus macaques lose the capacity to induce IL-8

**Figure 1:**
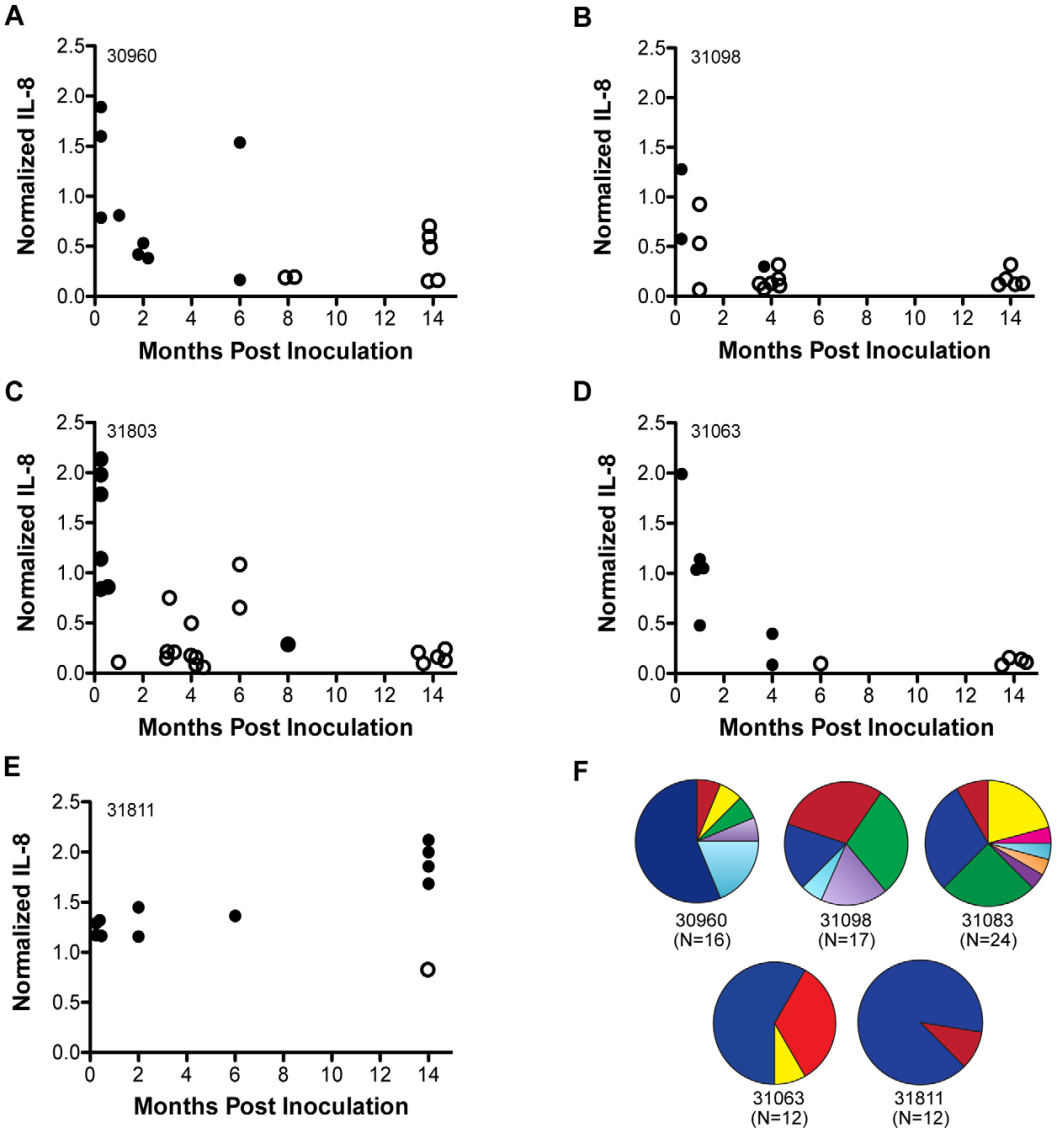
Loss of the capacity to induce IL-8 in *H. pylori* recovered from rhesus monkeys is associated with changes in the gene encoding CagY, an essential protein in the T4SS. (A–E) *H. pylori* was isolated from five rhesus macaques up to 14 months after experimental infection with *H. pylori* WT J166. Individual colonies were co-cultured with AGS cells, and ELISA was used to measure IL-8 levels, which were normalized to the WT J166 positive control. Each data point represents the results from a single colony. The capacity to induce IL-8 decreased over time in colonies recovered from four monkeys (A–D), but was largely unchanged in one (E). PCR-RFLP analysis showed that *H. pylori* colonies that lost the capacity to induce IL-8 were associated with a change in *cagY* (open circles), while those that maintained IL-8 induction typically had *cagY* that was indistinguishable from WT J166 (filled circles). Animal designation is shown in the upper left corner of each panel. (F) Output strains from each monkey were analyzed by *cagY* PCR-RFLP and compared to WT *H. pylori* J166 (dark blue) and to one another. Each pie chart represents all colonies recovered from one of the five monkeys (12–24 colonies/monkey); different colors represent different *cagY* variants.

*H. pylori* strains adapted to colonization of mice frequently lose the capacity to induce IL-8 and translocate CagA into gastric epithelial cells [20], [21], which are measures of a functional T4SS. The *cag*PAI is retained and the mechanism is unknown [21]. Since mice are not a natural host for *H. pylori*, we asked whether similar changes occur during infection of rhesus macaques, which are commonly infected with *H. pylori* that is indistinguishable by comparative genomic hybridization from that which infects humans [22]. Five rhesus monkeys were previously challenged with a single colony of wild type (WT) *H. pylori* J166 that has a functional *cag*PAI [23]. Multiple output colonies recovered from each monkey up to 14 months post inoculation (PI) were co-cultured with AGS gastric cells to determine their capacity to induce IL-8, which was normalized to the WT control strain. IL-8 induction resembled WT in bacteria recovered early after challenge, but decreased over time in 4 of 5 monkeys (Figure 1A–D). In one monkey, all but one bacterial colony induced IL-8 at levels≥WT, even after 14 months of colonization (Figure 1E). Selected rhesus output colonies that induced low IL-8 (designated rOut1 and rOut2) or high IL-8 (rOut3) in AGS cells were also tested in KATO III gastric cells. Similar results were obtained (Figure S1A). These results demonstrate that *H. pylori* infection of rhesus monkeys results in a population of strains that have lost the capacity to induce the pro-inflammatory cytokine, IL-8, though there are individual differences among animals. Since loss of T4SS function occurs in macaques as well as mice, yet differs among individuals, it may represent a physiologic accommodation to the gastric environment.

### Changes in the capacity for induction of IL-8 during colonization of macaques are associated with recombination in *cagY*

Systematic mutagenesis experiments have demonstrated that 15 genes on the *cag*PAI (*cagδ*, *cagγ*, *virB11*, *cagY*, *cagX*, *cagW*, *cagV*, *cagU*, *cagT*, *cagM*, *cagL*, *cagI*, *cagH*, *cagE*, *cagC*) are essential for *H. pylori* to fully induce IL-8 [14], [15]. In some strains, *cagA* is required as well [24]. To determine if change in one or more of these genes was responsible for loss of IL-8 induction during colonization of rhesus monkeys, we amplified and sequenced each of these genes from WT J166 and from a rhesus output strain (rOut1) that had lost the capacity to induce IL-8. Each of the 16 genes was identical between WT J166 and rOut1 with the exception of *cagY*, in which a 321 bp fragment was deleted.

Dot-plot analysis (Figure S2) demonstrated that, like in strains J99 and 26695 [18], [25], *cagY* in *H. pylori* J166 has a large number of direct repeats that are organized into a 5′ repeat region (FRR) and a middle repeat region (MRR), in which the 321 bp deletion in rOut1 was located. The large number of direct repeats in *cagY* could permit deletion or duplication of the intervening region with preservation of an open reading frame, and might alter the functionality of the *cag*PAI. Since high throughput DNA sequencing of *cagY* is difficult owing to its large size and repeat structure, we used PCR-RFLP to determine if recombination in *cagY* occurred during infection of rhesus monkeys, and if it was associated with altered capacity to induce IL-8. Figure S3A shows representative *cagY* PCR-RFLP patterns from WT J166 and rOut1-3, each of which is unique. Each monkey was colonized by multiple unique *cagY* variants with the exception of one (31811), in which all but one colony induced IL-8≥WT and had a *cagY* that was indistinguishable from that in WT J166 (Figure 1F). We next compared the *cagY* PCR-RFLP from all 81 output colonies with their capacity to induce IL-8, and asked if *cagY* was the same (solid circles) or different (open circles) from that of WT J166 (Figure 1). Among all monkey output colonies that had normalized IL-8 induction ≥1.0, 96% (23 of 24) had the same *cagY* PCR-RFLP fingerprint as WT J166, while only 25% (14 of 57) of colonies with IL-8 induction <1.0 showed the same fingerprint (Fisher's exact test, two-tailed, *P*<0.0001). Output strains in which *cagY* differed from WT J166 typically showed IL-8 induction similar to the mean (±SEM) of a *cagY* deletion mutant (0.29±0.04). Loss of IL-8 induction without an apparent change in *cagY* may sometimes occur due to the inability of PCR-RFLP to detect frameshift mutations that lead to premature stop codons in *cagY*, or to a change in other *cag*PAI genes, including *cagA*, which is essential for full induction of IL-8 in *H. pylori* J166 (Figure S4). To determine if changes in *cagY* might be due simply to frequent recombination during *in vitro* culture, WT *H. pylori* J166 was passaged daily for 5 weeks, and each week 6 individual colonies were isolated and examined by PCR-RFLP. Of the 30 clones tested, 28 (93%) showed the same RFLP pattern and similar mean (±SEM) IL-8 induction (0.91±.01) as WT J166; the two clones with a different *cagY* RFLP showed reduced induction of IL-8 (0.32±.00). Since loss of IL-8 induction and change in *cagY* were common during experimental infection but not during *in vitro* passage, these results suggest that *H. pylori* infection of rhesus macaques selects for allelic diversity in *cagY* that is associated with decreased capacity to induce IL-8.

### Recombination in *cagY* is sufficient to modify the induction of IL-8 and phosphorylation of CagA

**Figure 2:**
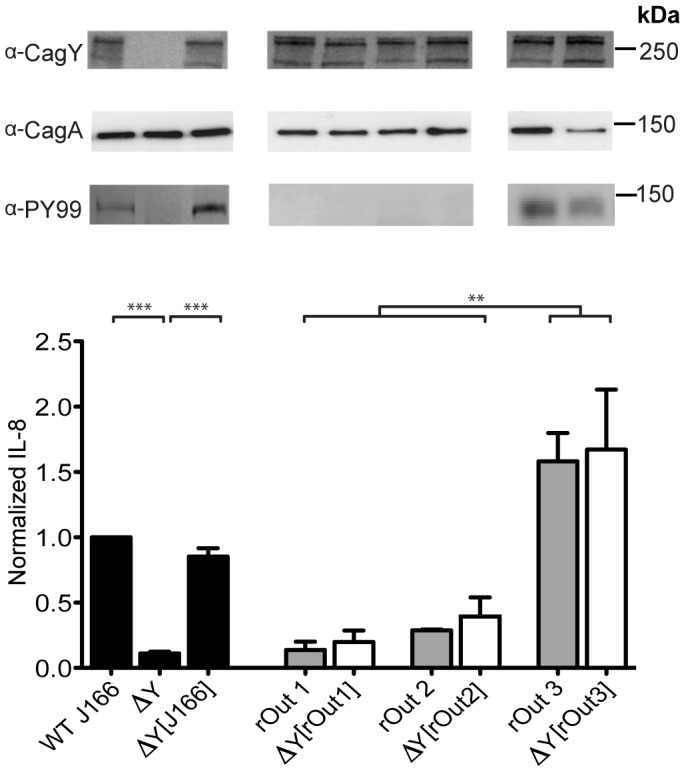
Recombination in *cagY* during infection of rhesus monkeys is sufficient to reduce the capacity of *H. pylori* to induce IL-8 and translocate CagA. Deletion of *cagY* (▵Y) from WT *H. pylori* J166 significantly reduced its capacity to induce IL-8 (mean ± SEM of 3 replicates), which was recovered when the chromosomal WT *cagY* allele was restored (▵Y [J166]) by complementation (black bars). Immunoblot showed that only the WT or ▵Y [J166] expressed CagY protein (α-CagY) and translocated CagA that was tyrosine phosphorylated (α-PY99). Two rhesus output strains with unique *cagY* alleles (rOut1, rOut2) lost the capacity to induce IL-8 (gray bars) and translocate CagA, although they expressed CagY. Replacement of ▵*cagY* with *cagY* from rOut1 (▵Y [rOut1]) or rOut2 (▵Y [rOut2]) recapitulated their failure to induce IL-8 induction (white bars) and translocate phosphorylated CagA. Similarly, complementation with *cagY* from an output strain (rOut3) that expressed a unique *cagY* but maintained the capacity to induce IL-8 (gray bar) and translocate CagA, also phenocopied its IL-8 induction and translocation of CagA. All strains expressed CagA (α-CagA), though only those that induced IL-8 had the capacity to translocate CagA that was tyrosine phosphorylated. Multiple bands in the CagY immunoblot could represent different transcription or translation products, or even protein fragments, but they are CagY-specific since they are absent in the *cagY* deletion mutant. **P<0.01; ***P<0.001.

Recombination in *cagY* might be associated with changes in IL-8, but not mechanistically linked to the function of the *cag*PAI. Therefore, we used contraselection [26], [27] to replace the *cagY* in WT J166 with the *cagY* gene from rOut1 or rOut2, each of which induced low IL-8 and had a unique *cagY* RFLP pattern. The *cagY* gene from streptomycin resistant J166 was deleted by homologous recombination with the *cat-rpsL* cassette (chloramphenicol resistant, dominant streptomycin susceptible), and then transformed with chromosomal DNA from either WT J166 (restoring the WT *cagY* allele) or one of the two rhesus output strains. Transformants that were chloramphenicol susceptible and streptomycin resistant (due to loss of the cassette), and had the appropriate *cagY* gene by PCR-RFLP and confirmed by full-length DNA sequence analysis, were then tested for induction of IL-8 and translocation of CagA. As expected, deletion of *cagY* in J166 markedly reduced IL-8 induction, and replacement of the WT *cagY* allele restored expression of CagY and induction of IL-8 (Figure 2). In contrast, replacement with *cagY* from rOut1 and rOut2, which induced low IL-8, did not restore IL-8 induction, even though the CagY protein was expressed. Although it was uncommon, we also identified a rhesus output strain (rOut3) that induced IL-8 at a level similar to WT J166, but had a unique *cagY* allele. As expected, replacement of the WT *cagY* allele with *cagY* from rOut3 maintained the capacity to induce IL-8. Only those strains that induced IL-8 were also capable of inducing CagA translocation and phosphorylation. These results demonstrate that recombination in *cagY* is sufficient to alter the functionality of the T4SS encoded by the *cag*PAI.

### Host immunity is required for *in vivo* selection of *cagY* variants that have lost the capacity to induce IL-8

**Figure 3:**
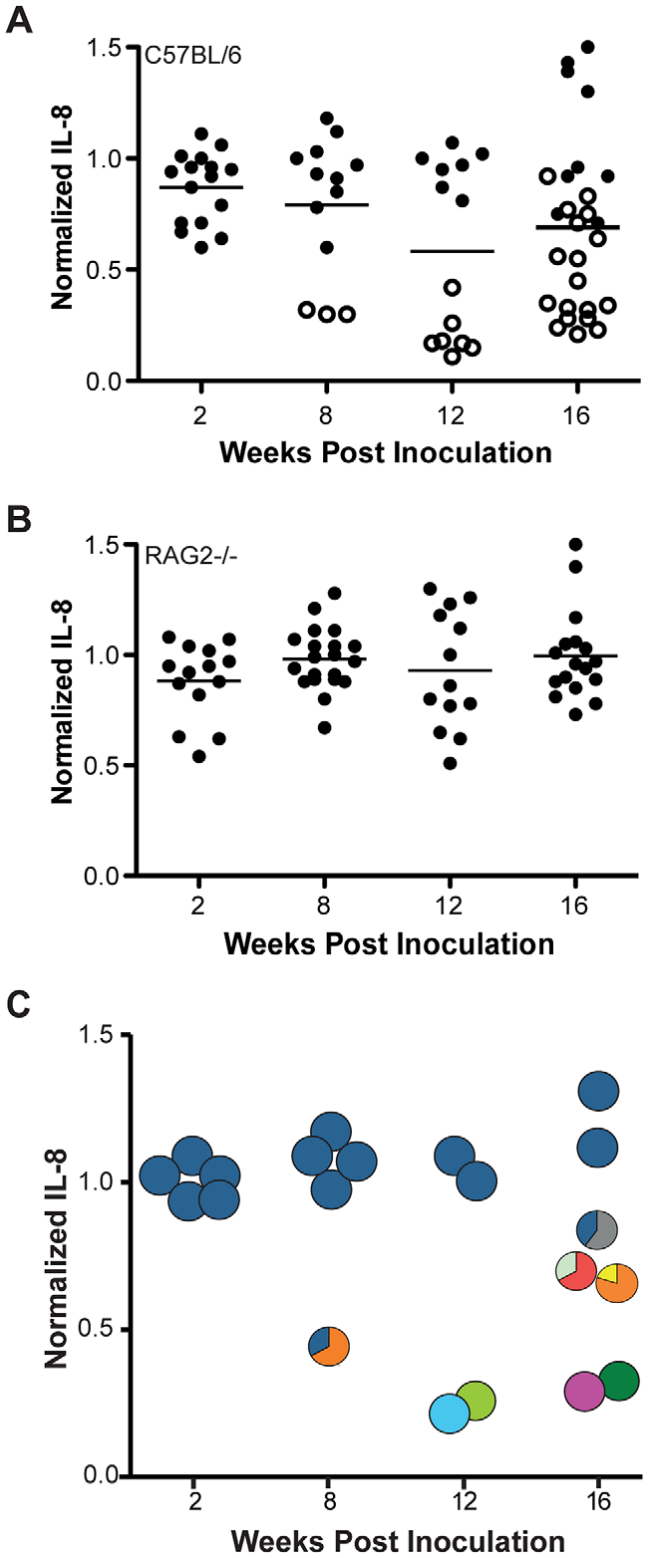
Loss of the capacity to induce IL-8 and change *cagY* during infection of mice is dependent on an intact host immune system. *H. pylori* was isolated from C57BL/6 WT (A) or RAG2−/− (B) mice (N = 3–6/time point) up to 16 weeks after experimental infection with *H. pylori* WT J166. Individual colonies (3–6/mouse) were co-cultured with AGS cells, and ELISA was used to measure IL-8 levels, which were normalized to the WT J166 positive control (line = mean). Each data point represents the results from a single colony. Induction of IL-8 in colonies isolated from WT mice was significantly lower than in RAG2−/− mice at 12 and 16 weeks PI (*P*<0.01). Changes in *cagY* (open circles) were detected by PCR-RFLP in 28 of 70 colonies from WT mice but in 0 of 64 colonies from RAG2−/− mice (Fishers exact test, *P*<0.0001). Output strains from WT C57BL/6 mice were analyzed by *cagY* PCR-RFLP and compared to WT *H. pylori* J166 (dark blue) and to one another (C). Each pie chart represents the unique *cagY* RFLP patterns identified in a single mouse from 2 to 16 weeks PI, and is positioned according to the mean IL-8 induction by colonies recovered from that mouse.

**Figure 4:**
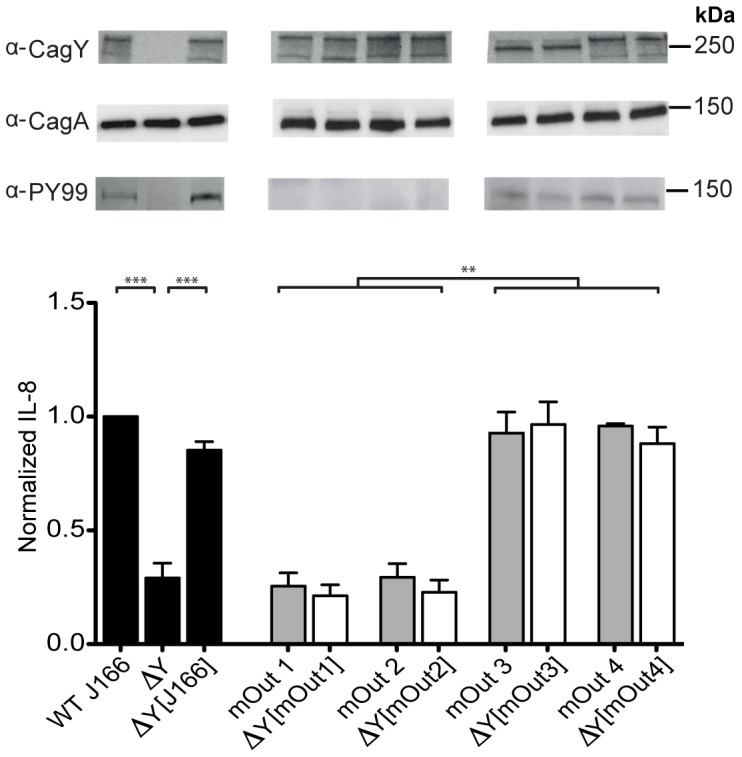
Recombination in *cagY* during infection of mice is sufficient to reduce the capacity of *H. pylori* to induce IL-8 and translocate CagA. Deletion of *cagY* (▵Y) from WT *H. pylori* J166 significantly reduced its capacity to induce IL-8 (mean ± SEM of 3 replicates), which was recovered when the chromosomal WT *cagY* allele was restored (▵Y [J166]) by complementation (black bars). Two output strains from C57BL/6 mice with unique *cagY* alleles (mOut1, mOut2) lost the capacity to induce IL-8 (gray bars) and translocate CagA, although they expressed CagY (α-CagY). Complementation of ▵*cagY* with *cagY* from mOut1 (▵Y [mOut1]) or mOut2 (▵Y [mOut2]) recapitulated their lack of IL-8 induction (white bars) and translocation of phosphorylated CagA (α-PY99). Similarly, replacement with *cagY* from two output strains (mOut3, mOut4) that expressed a unique *cagY* but maintained the capacity to induce IL-8 (gray bars) and translocate CagA, also phenocopied their IL-8 induction and translocation of CagA. All strains expressed CagA (α-CagA), though only those that induced IL-8 had the capacity to translocate CagA that was tyrosine phosphorylated. Multiple bands in the CagY immunoblot could represent different transcription or translation products, or even protein fragments, but they are CagY-specific since they are absent in the *cagY* deletion mutant. **P<0.01; ***P<0.001.

Identification of the direct repeat structure of *cagY* suggested that frequent in-frame recombination events may be a mechanism of antigenic variation to avoid the host adaptive immune response [28]. To test this hypothesis, we inoculated WT *H. pylori* J166 into WT C57BL/6 and RAG2−/− mice, which do not have functional B or T cells and develop little or no gastric inflammation after *H. pylori* infection [29]. *H. pylori* colonization levels were approximately 10-fold higher in RAG2−/− mice compared to WT mice (Figure S5A). Similar to the results in rhesus monkeys, bacteria recovered from WT mice resembled input *H. pylori* early after challenge (Figure 3A). However, at 12 and 16 weeks PI, bacteria from WT mice showed a significant loss in IL-8 induction (*P*<0.01) and change in *cagY* (*P*<0.001) compared to colonies from RAG2−/− mice, which uniformly resembled WT J166 in IL-8 induction and showed no changes in *cagY* by RFLP analysis (Figure 3B). We next replaced the *cagY* allele in WT *H. pylori* J166 with that from mouse output strains that changed *cagY* and either lost (mOut1 and mOut2) or maintained (mOut3 and mOut4) IL-8 induction in AGS cells, which was confirmed in KATO III cells (Figure S1B). Similar to the results with rhesus output strains (Figure 2), induction of IL-8 and phosphorylation of CagA in mouse output strains were phenocopied when their *cagY* allele was used to replace that in WT J166 (Figure 4). Interestingly, the bacterial population within each individual mouse was relatively homogenous, showing either WT levels of IL-8 and *cagY* indistinguishable from input, or low IL-8 and one or at most two unique *cagY* variants (Figure 3C). These experiments demonstrate that CagY-mediated change in function of the *H. pylori* T4SS is dependent on an intact host immune system.

### *cagY* variants that fail to induce IL-8 and translocate CagA do not induce expression of NF-κB

**Figure 5:**
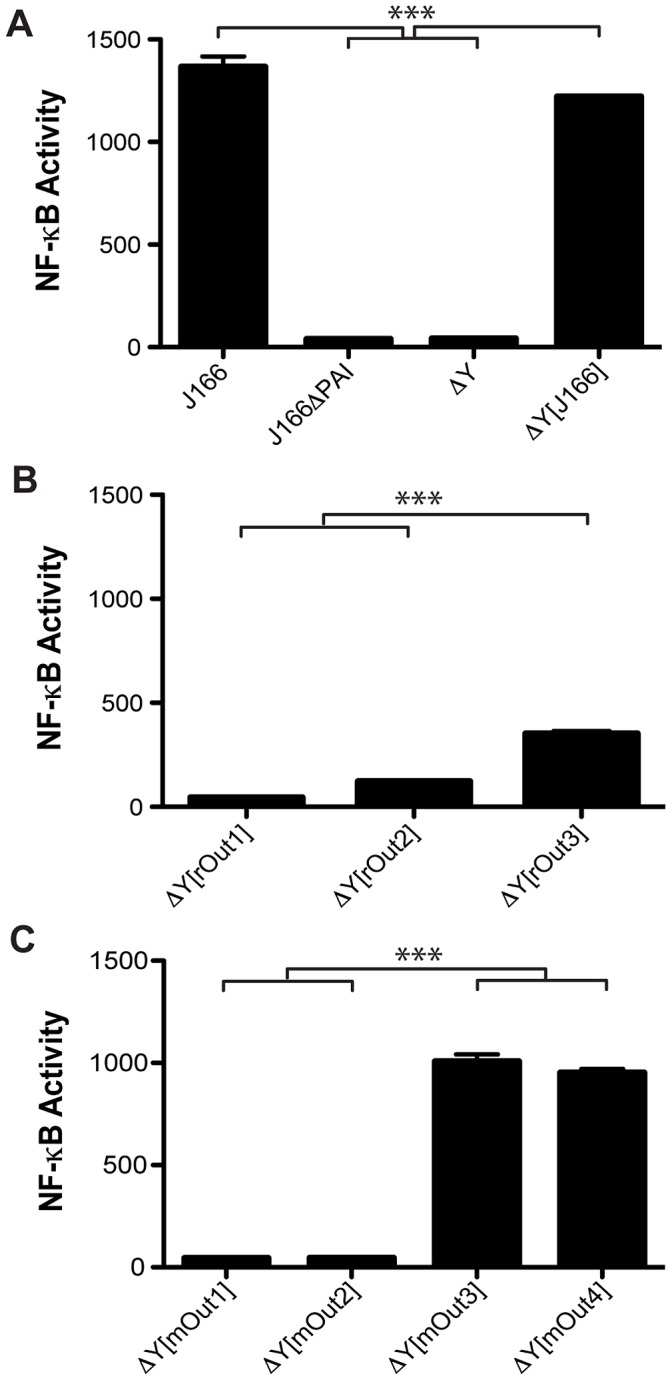
*cagY* variants that fail to induce IL-8 and translocate CagA do not induce expression of NF-κB. (A) Co-culture of *H. pylori* with AGS cells stably transformed with a reporter plasmid demonstrated that activation of NF-κB was seen in WT J166 but not in a strain with a deletion of the *cag*PAI (▵PAI). Reintroduction of J166 *cagY* into a *cagY* deletion mutant restored NF-κB activation. Introduction of *cagY* from monkey (B) or mouse (C) output strains showed that increased NF-κB activation compared to ▵*cagY* (▵Y) or ▵PAI was seen only in strains bearing a *cagY* allele that was competent for induction of IL-8 and translocation of CagA (rOut3, mOut3, mOut4). ***P<0.001.

Although *H. pylori*-induced signaling cascades in host cells are complex and poorly understood, it is clear that T4SS-mediated pro-inflammatory responses are dependent upon activation of the transcription factor, NF-κB [30]. Therefore, we examined NF-κB activation using an AGS cell line stably transfected with a luciferase reporter construct. AGS cells were co-cultured with WT J166 or isogenic J166 strains encoding *cagY* from monkey or mouse output strains. Phorbol myristate acetate (PMA) and deletions in the entire *cag*PAI or in *cagY* were used as positive and negative controls, respectively. Similar to strains with a deletion in *cagY* or the entire *cag*PAI, *cagY* variants that failed to induce IL-8 and translocate CagA (rOut1,2; mOut1,2) also failed to activate NF-κB (Figure 5). In contrast, introduction of *cagY* alleles from strains that induced IL-8 and translocated CagA (rOut3; mOut3,4) showed significantly increased activation of NF-κB, though rOut3 did not achieve a level similar to WT J166. These results suggest that *cagY* mediated alterations in T4SS function is mediated largely by NF-κB.

### Variants in CagY demonstrate a modular change in structure

**Figure 6:**
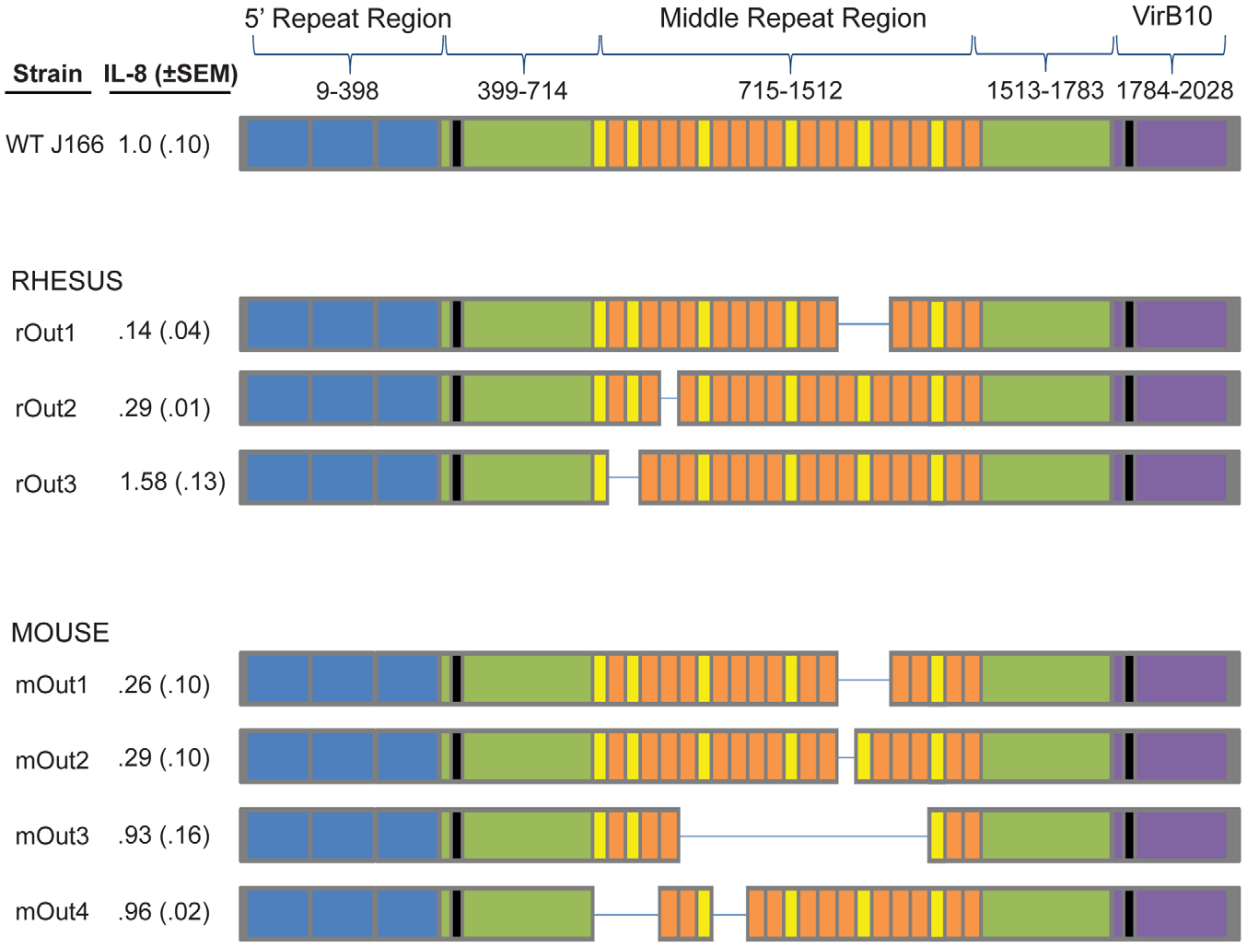
*H. pylori* colonization of rhesus monkeys and mice is associated with changes in the motif structure of the CagY middle repeat region. As in strains J99 and 26695 [19], the predicted amino acid sequence of CagY in WT *H. pylori* J166 is organized into a 5′ repeat region (residues 9–398), a 3′ region orthologous to VirB10 (residues 1784–2028), and a middle repeat region (residues 715–1512) that is composed of a series of B motifs (yellow) that bracket one to four A motifs (orange). Passage of WT J166 in rhesus monkeys and in mice results in some strains that lose one or more A or B motifs, which is sometimes sufficient to reduce the capacity to induce IL-8 (rOut1 and rOut2; mOut1 and mOut2) and other times is not (rOut3; mOut3 and mOut4).

Previous analysis of 14 full-length CagY sequences in the NCBI non-redundant protein data base suggested that the MRR is organized into two α-helical principal motifs, which occur in tandem arrays of one to six 38–39 residue A motifs flanked by single copies of a 31 residue B motif [19]. Both principal motifs are made up of three distinct submotifs, which remain invariant in their order. This annotation suggests that CagY variants that are selected *in vivo* are likely a result of duplication or deletion of principal motif segments, without compromising the underlying submotif composition. To examine this, we first identified the A and B principal amino acid motifs in the CagY MRR of WT *H. pylori* J166. Similar to other *H. pylori* strains previously described [19], the CagY MRR of *H. pylori* J166 is organized into six tandem arrays of one to four A motifs flanked by B motifs (Figure 6). We next examined the motif structure of CagY from rhesus (rOut1-3) and mouse (mOut1-4) output strains that were previously characterized (Figures 2 and 4). All output strains from monkeys and mice with variant *cagY* alleles had lost one or more A or B motifs, though there were multiple CagY motif structures associated with the same IL-8 phenotype. One output strain each from monkey (rOut1) and from mouse (mOut1), which had both lost the capacity to induce IL-8, had identical motif structures. Interestingly, loss of a single A motif was sufficient to markedly reduce IL-8 induction (rOut2), while loss of 14 A and B motifs (mOut3), representing a reduction in predicted size from 233 kDa to 175 kDa, was not. Although we were unable to identify a motif pattern associated with the IL-8 phenotype, these results suggest that CagY function is based on a higher order structure and not on any critical motif within the MRR.

### The T4SS pilus is expressed in *H. pylori* strains with functional and non-functional *cagY* alleles

**Figure 7:**
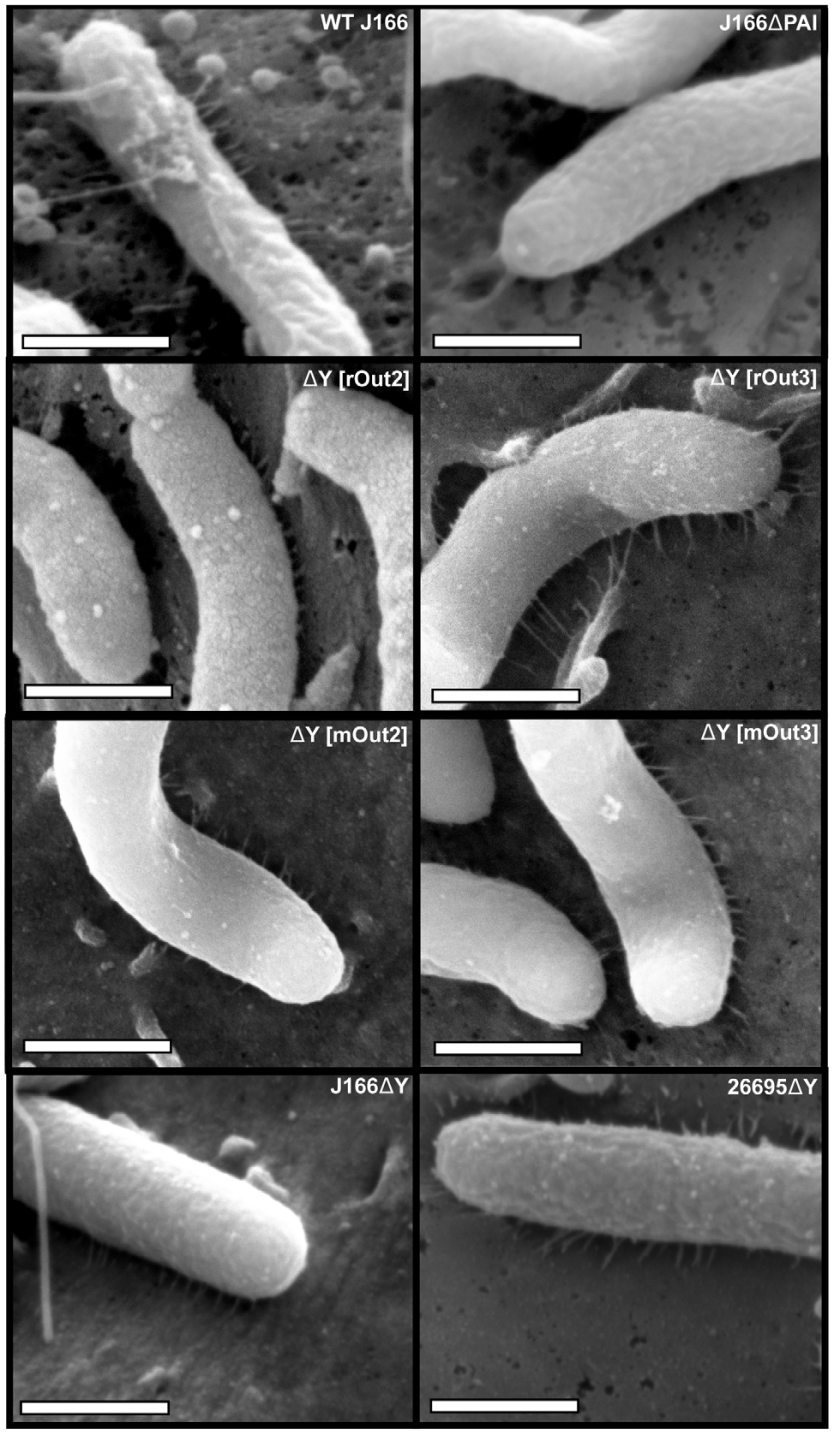
Changes in the motif structure of the CagY middle repeat region that alter the function of the *cag*PAI do not affect expression of T4SS pili on the bacterial surface. *H. pylori* was co-cultured with AGS gastric cells at an MOI of 100∶1 and imaged by FEG-SEM. T4SS pilus structures were readily apparent in the WT *H. pylori* J166 but not in the *cag*PAI deletion mutant (J166▵*cag*PAI). T4SS pili were also observed in *H. pylori* J166 in which the WT *cagY* allele was replaced with that from output strains with a functional (rOut3, mOut3) or a non-functional (rOut2 mOut2) *cag*PAI. Pili were also seen in *H. pylori* strains J166 and 26695 with deletions in *cagY*. Magnification bars indicate 500 nm.

Output colonies that have variant *cagY* alleles and no longer induce IL-8 still express CagY (Figures 2 and 4), but they might not make T4SS pili, or the pili might have altered structural features. To test this possibility, we used field emission scanning electron microscopy (FEG-SEM) to image *H. pylori* strains co-cultured with AGS cells. As expected, WT J166 but not a *cag*PAI deletion mutant produced pilus-like structures (Figure 7). This is consistent with previous studies demonstrating that the *cag*PAI is essential for the formation of a T4SS [2], [15], [31], [32]. Pili of similar dimensions were previously reported to be present in WT strain 26695, but absent in *H. pylori* 26695 with deletions of *cagT*, *cagE*, *cagL*, and *cagI*, all of which are required for a functional T4SS [15]. Using this imaging approach, we examined isogenic strains of *H. pylori* J166 in which the *cagY* gene had been replaced with alleles from strains that did (rOut3, mOut3) or did not (rOut2, mOut2) induce IL-8 and translocate CagA (Figures 2 and 4). Regardless of *cag*PAI functionality, all strains made pilus structures (Figure 7). Although the pili were less prominent on some strains that had defects in T4SS function, we were unable to identify a reproducible association between *cag*PAI function and quantitative measures of pilus number or morphology (Table S1).

**Figure 8:**
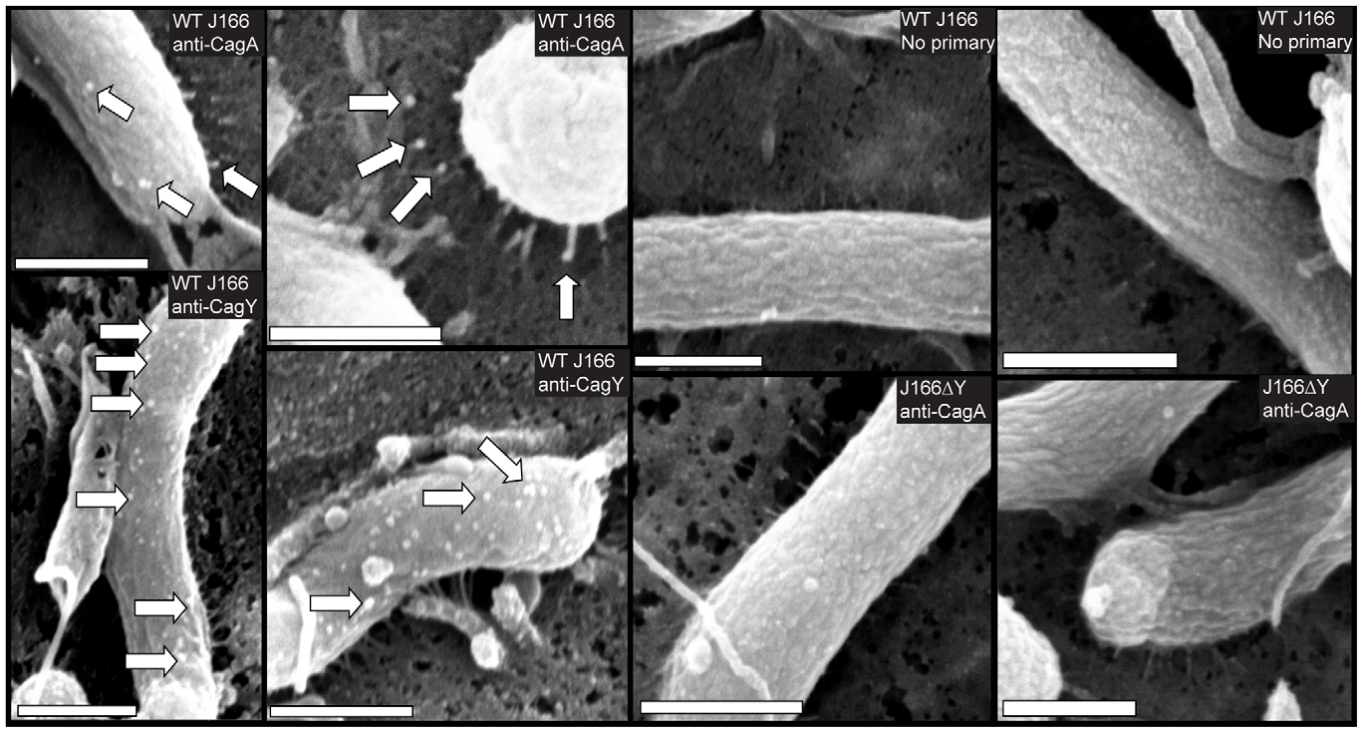
CagY decorates the *H. pylori* bacterial surface but is not associated with T4SS pili. *H. pylori* was co-cultured with AGS gastric cells at an MOI of 100∶1, incubated with antibodies to the CagY MRR or CagA, and imaged by FEG-SEM in the environmental mode. CagY was detected on the bacterial surface of the WT strain but was not associated with pili. CagA was detected both on the bacterial surface and in close approximation to the tips of the pili of the WT strain. There was markedly reduced CagA labeling on the surface of ▵*cagY* mutant strain compared to the WT strain. No staining was seen when primary antibody was omitted. Pili are sometimes not as well visualized and more often appear broken in these images compared to Figure 7 due to the lack of metal coating and more frequent washes. Magnification bars indicate 500 nm.

Pilus structures were also seen in *H. pylori* J166 with a deletion of *cagY* (Figure 7); similar results were obtained with a *cagY* deletion in *H. pylori* strain 26695 (Figure 7). To investigate the cellular localization of CagY, we performed immunogold SEM using antibody to the CagY MRR to stain *H. pylori* co-cultured with AGS cells. Antibody to CagA was used as a positive control. Although CagY label was seen scattered over the bacterial cells in WT *H. pylori*, no staining was found on or near the pilus structure (Figure 8). In contrast, CagA was identified both on the cell surface and closely approximated to the tips of pili in WT *H. pylori*, which has been reported previously [2]. CagA was not detected in association with pili in a *cagY* deletion mutant, in which the T4SS is not functional, and there was markedly reduced CagA labeling on the surface of the *cagY* mutant bacteria compared to WT (Figure 8). The absence of detectable CagY in association with pili is consistent with the finding that a *ΔcagY* mutant produces pili that are indistinguishable from those in the WT strain. Together, the EM results suggest that the loss of function that occurs with changes in CagY results from a functional change in the T4SS without any detectable structural defect in the T4SS pilus.

### *H. pylori* SS1, the commonly used mouse-adapted strain that does not induce IL-8 or translocate CagA, has a non-functional CagY

**Figure 9:**
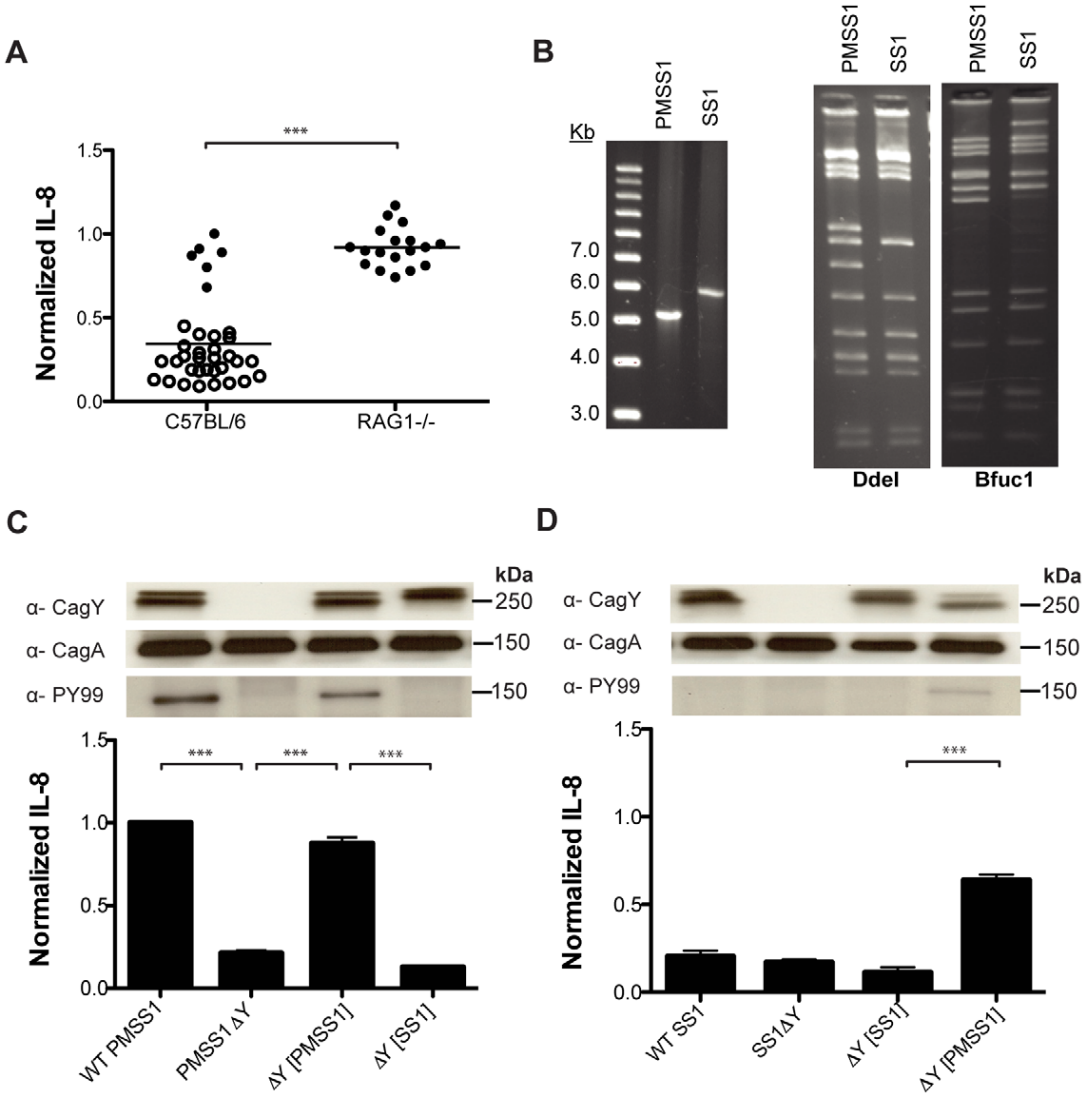
Mouse adapted *H. pylori* strain SS1 expresses a CagY that is not functional for induction of IL-8 or translocation of CagA. (A) *H. pylori* was isolated from C57BL/6 WT or RAG1−/− mice (N = 3–6/time point) 8 weeks after experimental infection with *H. pylori* PMSS1. Individual colonies (3–6/mouse) were co-cultured with AGS cells, and ELISA was used to measure IL-8 levels, which were normalized to the PMSS1 positive control (line = mean). Each data point represents the results from a single colony. Induction of IL-8 in colonies isolated from WT mice was significantly lower than in RAG1−/− mice, and was associated with changes in *cagY* PCR-RFLP (open circles). (B) *cagY* in *H. pylori* strain SS1 is larger than that in the progenitor strain PMSS1, and has a different fingerprint on PCR-RFLP. (C) Deletion of *cagY* from WT *H. pylori* PMSS1 reduced the induction of IL-8 and eliminated translocation of CagA, which were recovered when the WT PMSS1 *cagY* gene was restored (▵Y[PMSS1]. However, replacement of the PMSS1 *cagY* gene with that from *H. pylori* SS1 (▵Y [SS1]) showed reduced levels of IL-8 and no CagA translocation. (D) WT *H. pylori* SS1 showed little induction of IL-8 and no CagA translocation, and it was unaffected by deletion of *cagY* or restoration of the WT SS1 *cagY* allele. However, replacement of the WT SS1 *cagY* allele with that from PMSS1 markedly increased IL-8 induction and CagA translocation, though not to the level of PMSS1. All assays represent the mean ±SEM of 3 replicates. **P<0.01; ***P<0.001.

Studies of *H. pylori* pathogenesis were long hampered by the inability of investigators to successfully colonize mice. Since the difficulty was attributed primarily to *H. pylori* strain differences, mouse-adapted strain SS1 was derived, which has become the standard for animal experimentation [33]. However, it was later realized that *H. pylori* SS1 did not induce IL-8 or translocate CagA [20], [21], despite having an intact *cag*PAI detected by microarray [34]. The reason for this is unknown. It was recently reported that the original human isolate, designated pre-mouse SS1 (PMSS1), does have a functional *cag*PAI [35]. We therefore hypothesized that SS1 had undergone recombination in *cagY* during mouse passage that eliminated its capacity to induce IL-8 and translocate CagA. To test this hypothesis, we first inoculated PMSS1 into WT C57BL/6 and RAG1−/− mice, and examined IL-8 induction and *cagY* RFLP in colonies recovered 8 weeks PI. Similar to the results with strain J166 (Figure 3), colonies from WT but not RAG1−/− mice showed loss of IL-8 induction that was associated with recombination in *cagY* (Figure 9A). These results are consistent with a previous report demonstrating loss of T4SS function after challenge with PMSS1 in adult but not neonatal mice, which control effector T cell responses by *H. pylori*-specific regulatory T cells [35].

The *cagY* allele in SS1 is much larger than that in PMSS1 and has a markedly different PCR-RFLP pattern (Figure 9B). To determine if the increase in size of *cagY* was responsible for loss of a functional T4SS in SS1, we used contraselection to exchange the *cagY* genes between PMSS1 and SS1, and tested the strains for induction of IL-8 and translocation of CagA. As expected, *H. pylori* PMSS1 induced IL-8 and translocated CagA (Figure 9C), while SS1 did not (Figure 9D), although both expressed CagA and CagY. However, when *cagY* from SS1 was introduced into PMSS1, it could no longer translocate CagA or induce IL-8 (Figure 9C), indicating that the SS1 CagY was not functional. Interestingly, when *cagY* from PMSS1 was introduced into SS1, CagA translocation and IL-8 induction increased, but not to the level of PMSS1 (Figure 9D), suggesting that alteration in *cagY* is not the only defect in the T4SS of SS1. Together, these results suggest that *H. pylori* SS1 underwent recombination in *cagY* during mouse passage, which eliminated the functionality of the T4SS, reduced its inflammatory capacity, and enhanced its colonization of mice.

### *In vivo* recombination in CagY can also restore the capacity to induce IL-8

**Figure 10:**
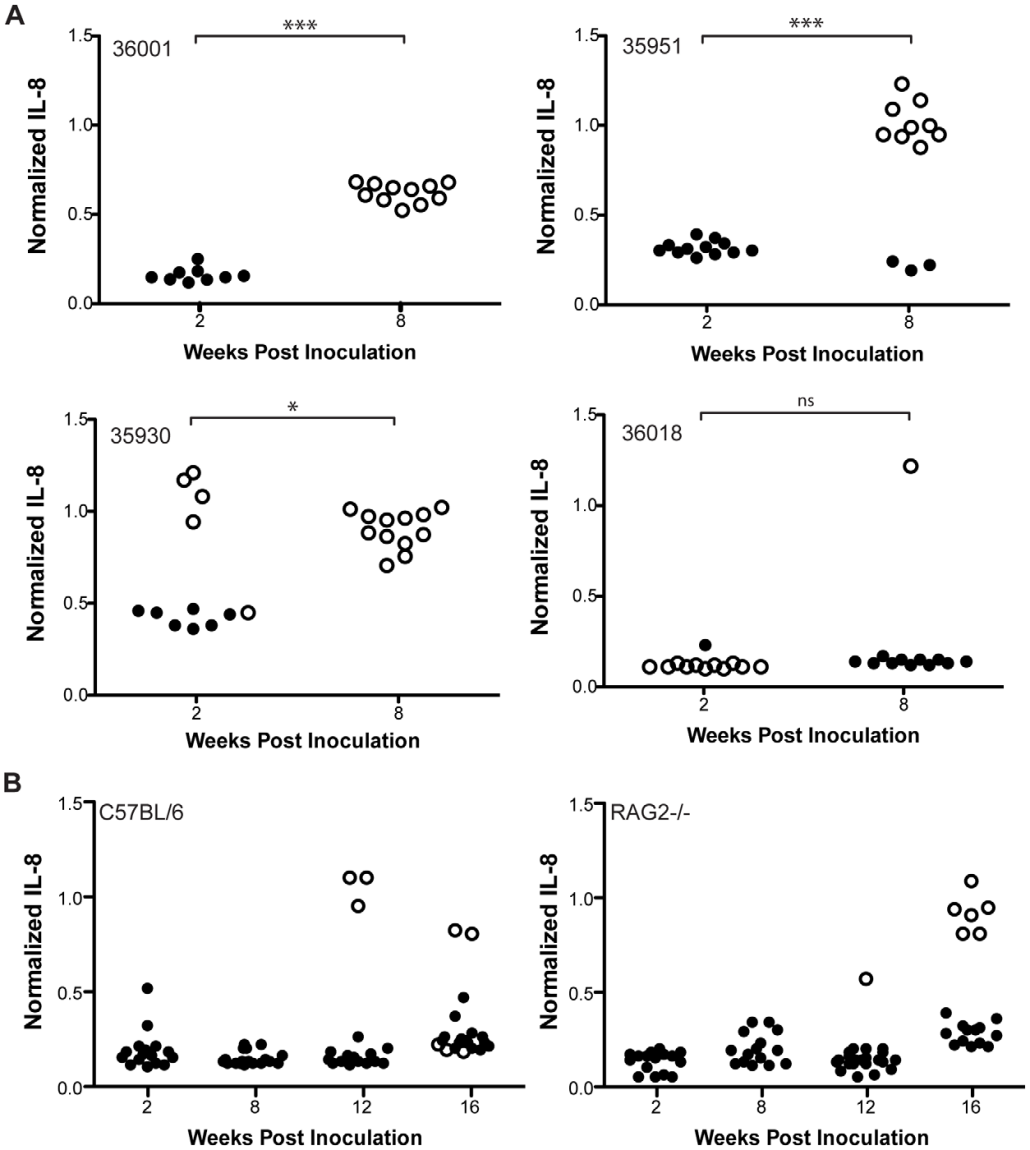
Recombination of *cagY* during infection of rhesus macaques and mice can also restore the capacity to induce IL-8. Rhesus macaques and mice were inoculated with mOut2, which does not induce IL-8 or translocate CagA. Single colony isolates were recovered and tested for induction of IL-8 and compared to mOut2 by *cagY* PCR-RFLP. (A) Colonies from three monkeys (36001, 35951, 35930) showed significantly increased capacity to induce IL-8 at 8 weeks compared to 2 weeks PI, which was associated with changes in *cagY* RFLP. The fourth monkey (36018) was colonized with a mixture of *cagY* genotypes that induced low IL-8 similar to mOut2. (B) Colonies recovered from WT and RAG2−/− mice typically induced low IL-8 similar to input mOut2, with no change in *cagY*. *P<0.05; ***P<0.001.

Recombination in *cagY* could be a mechanism by which *H. pylori* modulates rather than evades the host inflammatory response. If so, *in vivo cagY* recombination might sometimes confer an increase in the function of the T4SS, and enhance rather than reduce *H. pylori* inflammatory potential. To address this hypothesis, we undertook experiments to investigate possible alterations in *cagY* that might occur if animals were challenged with *H. pylori* mOut2, which had undergone *cagY* recombination that eliminated function of the T4SS (Figure 4). As a first step, to exclude the possibility that additional mutations could have occurred in mOut2 that conferred loss of T4SS function, we used contraselection to replace the *cagY* in mOut2 with that from WT J166. The results demonstrated that replacement of *cagY* in this strain with *cagY* from WT J166 was sufficient to restore induction of IL-8 in mOut2 (Figure S6). In three of four monkeys infected with mOut2 (36001, 35951, 35930), most colonies recovered two weeks after challenge resembled the input, with low IL-8 induction and the same *cagY* PCR-RFLP (Figure 10A). However, by eight weeks there was a significant increase in the capacity to induce IL-8 that was accompanied by changes in the *cagY* RFLP. One of these three monkeys (36001) was sampled repeatedly up to 24 weeks post inoculation; all output colonies recovered 8 weeks or more PI induced IL-8 and expressed a *cagY* that differed from that in mOut2 (Figure S7). A fourth monkey (36018) was colonized with a mixed population of *cagY* variants, but nearly all induced low IL-8 similar to that of the challenge strain. We next infected C57BL/6 WT and RAG2−/− mice with mOut2, and analyzed IL-8 induction and *cagY* RFLP up to 16 weeks PI. Similar to infection with WT J166, colonization density of mOut2 was greater in RAG2−/− mice than in C57BL/6 mice (Figure S5B). In general, strains recovered from both WT and RAG2−/− mice induced low IL-8 similar to the input mOut2, with no change in *cagY* (Figure 10B). A few colonies from both WT and RAG2−/− mice showed increased IL-8, which was accompanied by a change in *cagY*. Strains from mice and monkeys that recovered IL-8 induction showed novel *cagY* RFLP fingerprints that did not revert to WT J166. These results demonstrate that *in vivo* recombination in *cagY* can either eliminate or restore the function of the T4SS encoded on the *H. pylori cag*PAI. Since CagY that confers a non-functional T4SS appears stable in mice, modulation may be driven more by inflammation rather than adaptive immune responses.

## Discussion

The capacity to evade or circumvent host defense is considered a signature of pathogenic bacteria that distinguishes them from closely related commensals [36]. The mechanisms by which this occurs are varied, and they include elaboration of toxins that inhibit the function of immune cells, iron sequestration, antigenic variation of surface structures, intracellular invasion, and inducing host expression of immunosuppressive cytokines, to name just a few. But bacterial pathogens not only avoid host immune responses, they also sometimes exploit them. This is perhaps best understood for infection with *Salmonella enterica* serotype Typhimurium, where the T3SS-dependent host inflammatory response is required for colonization of mice [37]. Inflammation generates tetrathionate, an electron acceptor that can be used by *S.* Typhimurium but not by competing microbiota [38]. Inflammation also induces epithelial cells to express lipocalin-2 and calprotectin, which sequester iron and zinc from the gut microbiota but not from *S.* Typhimurium because it expresses specialized high affinity metal transporters [39], [40]. Thus, from the bacterial point of view, the host inflammatory response can be both deleterious and advantageous.

The hallmark of infection with *H. pylori* is chronic active gastritis comprised of polymorphonuclear leukocytes together with Th1, Th17, and Treg CD4+ lymphocytes [41]. The *cag*PAI is central to the inflammatory response because *H. pylori* strains bearing the *cag*PAI are more often associated with clinical disease in humans, rather than asymptomatic infection. These epidemiologic observations are supported by studies showing that strains harboring isogenic deletions within the *cag*PAI cause less gastritis and precancerous pathology in animal models than do strains with an intact *cag*PAI [35], [42], [43]. Yet from the bacterial perspective, the *cag*PAI has mixed effects. On the one hand, enhanced inflammation induced by the T4SS partially controls the infectious burden and presumably decreases transmission and therefore fitness. On the other hand, T4SS-mediated injection of CagA enhances the fitness of *H. pylori* by altering epithelial cell polarity and increasing bacterial iron acquisition, which permits it to grow on the apical epithelial cell surface [7], [44]. Here we demonstrate that *H. pylori* has evolved a novel solution to this dilemma, in which *cagY*, an essential component of the T4SS, has highly repetitive DNA elements that undergo rearrangements that can change the functionality of the *cag*PAI. These rearrangements may occur in vivo, but they are likely also present in the bacterial inoculum, since we could identify *cagY* variants relatively easily *in vitro* (2 of 30 clones examined). Although we have not formally identified recombination as the mechanism (e.g., horizontal gene transfer is possible), this seems most likely given the high frequency of repetitive elements within the *cagY* gene. We propose that *cagY* is a sort of contingency locus [45] that generates diversity at the population level and enhances bacterial fitness by allowing adaptation to changing conditions that may be found within one host or during transmission to another.

The most obvious pressure that may select for variant *cagY* alleles is the host adaptive immune response. Earlier studies suggested that the repeat structure of *cagY* represented a mechanism for antigenic variation to evade adaptive immunity [18], which is consistent with our finding that variant *cagY* alleles develop during colonization of WT but not RAG−/− mice (Figures 3, 9). However, strains recovered from monkeys and WT mice infected with *H. pylori* J166 sometimes maintained the *cagY* of the input strain, even after prolonged colonization when adaptive immunity would be fully developed (Figures 1, 3). Moreover, humans chronically colonized with *H. pylori* do not mount a serum immune response to CagY [18]. Thus, avoiding adaptive immunity may not be adequate to explain our results. An alternative hypothesis is that CagY variants serve not to evade the host immune response, but rather to “tune” it so as to establish the optimal homeostatic conditions of inflammation under which *H. pylori* is most fit. This hypothesis is supported by our finding that infection of monkeys and mice can select *H. pylori* strains with either loss of function (Figures 1,3,9) or gain of function (Figure 10A) in the T4SS, and the observation that the *cagY* genotype is relatively stable in WT mice when it confers a non-functional T4SS (Figure 10B). Finally, the very fact that many functional and non-functional variants of CagY arise *in vivo* by recombination, suggests that inflammation must be more advantageous to the bacterium in some situations than in others. Studies in humans have sometimes identified patients with mixed populations of *cag*PAI+ and *cag*PAI− strains [46]. Some have suggested that there is in fact a dynamic equilibrium between *cag*PAI+ and *cag*PAI− strains, creating a sort of *H. pylori* quasispecies, where some PAI variants may be better suited for transmission to a new host, and others better adapted for chronic persistence [47]. *cag*PAI+ strains isolated from an individual patient may also differ markedly in functionality of the T4SS [48], which might be explained by variations in the motif structure of CagY, but could also arise from mutations in other *cag*PAI genes. However, given the high frequency of *cagY* recombination, it seems likely that this mechanism is a much more common strategy by which *H. pylori* modulates its capacity to induce inflammation than is, for example, frameshift mutation, or gain or loss of the entire *cag*PAI.

There may also be differences in the relative fitness of *H. pylori* strains with a functional or a non-functional T4SS, depending on the inflammatory response of an individual host. When infected with WT *H. pylori* J166, most monkeys selected for loss of function in the T4SS, though one did not, even after 14 months of colonization (Figure 1). Similarly, when infected with mOut2, which has a non-functional T4SS, most monkeys selected for strains with a gain of function, but one did not (Figure 10A). Interestingly, in the one monkey available for long-term follow up, all strains recovered up to 24 weeks PI continued to induce IL-8 (Figure S7). Individual differences in strains recovered from outbred macaques may reflect host polymorphisms in the inflammatory response to *H. pylori*, which are well known to exist in humans and to have important clinical consequences [49]. Differences were also seen in individual WT C57BL/6 mice, which sometimes had persistent colonization with WT J166, even after prolonged infection when most mice selected for non-functional *cagY* variants (Figure 3). At first glance this is surprising, since inbred C57BL/6 mice are usually thought to be genetically identical. However, infection of mice with *Helicobacter* can yield both a resistant (low bacterial load, severe pathology, extensive CD4+ T cell infiltration, high IFN-γ) and a tolerant phenotype [50], so inbred mice may in fact be more genetically diverse than is usually thought [51], [52]. If inflammation is critical to the *H. pylori* lifestyle, yet is variable among hosts, modulation of T4SS function by recombination in *cagY* may provide a flexible mechanism to colonize and adapt to heterogeneous populations.

Strains expressing variant *cagY* alleles with loss of T4SS function are indistinguishable from a *cag*PAI or *cagY* KO in their IL-8 induction and CagA phosphorylation, which suggests that they are defective in translocation of CagA and peptidoglycan. Structural and functional studies of the VirB10 orthologue in other Gram-negative bacteria provide some basis for speculation on potential mechanisms by which this might occur. Cryo-EM and crystallography studies of the T4SS encoded by the conjugative plasmid pKM101 showed that VirB10 assembles with VirB7 and VirB9 to form the outer surface of a core complex that spans the inner and outer bacterial membranes [17], [53]. The C-terminus portion of CagY that is homologous to VirB10 also forms a complex with the *H. pylori* VirB9 orthologue (CagX) [16], [54]. Similar to the energy coupling protein TonB, VirB10 in *A. tumefaciens* undergoes an energy dependent conformational change that is required for complex formation with VirB7 and VirB9, and subsequent delivery of the T-DNA substrate [55]. Recently a mutation has been identified in VirB10 from *A. tumefaciens* that confers a secretion system defect and regulates substrate passage across the bacterial outer membrane [56]. Hence, one mechanism by which CagY variants might alter function of the *H. pylori* T4SS is by gating the transfer of CagA, peptidoglycan, or other bacterial effectors across the host cell membrane.

Changes in the CagY MRR might also affect T4SS function by altering the binding to β1 integrins, which is essential for CagA translocation and signaling [1], [2]. A previous study suggested that the CagY MRR decorates the T4SS pilus [31]; another reported that pili are not observed after deletion of *cagY*, though the data were not shown [2], [32]. Changes in the modular structure of the MRR might affect T4SS function, either directly or by changing the integrin binding of other T4SS components required for pilus assembly [15]. However, we failed to find evidence of the CagY MRR on the surface of the T4SS pili, and no differences in pilus morphology were observed after deletion of *cagY*. Moreover, yeast two-hybrid studies suggest that β1 integrin binding occurs only with the CagY C-terminus [1], which is the region with homology to the *A. tumefaciens* VirB10 that spans the inner and outer bacterial membrane, However, extrapolation from studies of *A. tumefaciens* may be limited, because the predicted molecular mass of *H. pylori* CagY is 220 kDa, much larger than the predicted 45 kDa VirB10 from *A. tumefaciens*, which does not contain a region orthologous to the *H. pylori* MRR. For the moment, these inconsistencies remain unresolved.

In conclusion, we have identified a functional plasticity in the *H. pylori* T4SS. We propose that immune-driven host selection of rearrangements in CagY modulates the function of the *H. pylori* T4SS and “tunes” the host inflammatory response so as to maximize persistent infection. Future studies should address the mechanism by which CagY recombination alters T4SS signaling, and identify the immune effectors that select CagY variants.

## Materials and Methods

### Ethics statement

All animal experiments were performed in accordance with NIH guidelines, the Animal Welfare Act, and U.S. federal law. All experiments were carried out at the University of California, Davis under protocol #15597 approved by U.C Davis Institutional Animal Care and Use Committee (IACUC), which has been accredited by the Association of Assessment and Accreditation of Laboratory Animal Care (AAALAC). All animals were housed under these guidelines in an accredited research animal facility fully staffed with trained personnel.

### *H. pylori* strains and culture

*H. pylori* strain J166 has a functional *cag*PAI and colonizes both mice [27] and rhesus macaques [42]. *H. pylori* SS1 is a mouse-adapted derivative [33] of strain PMSS1, which is a human clinical isolate that has a functional *cag*PAI and also colonizes mice [35]. All *H. pylori* plate cultures were performed on brucella agar (BBL/Becton Dickinson, Sparks, MD) supplemented with 5% heat-inactivated newborn calf serum (Invitrogen, Carlsbad, CA) and either ABPNV (amphotericin B, 20 mg/liter; bacitracin, 200 mg/liter; polymyxin B, 3.3 mg/liter; nalidixic acid, 10.7 mg/liter; vancomycin, 100 mg/liter) or TVPA (trimethoprim, 5 mg/liter; vancomycin, 10 mg/liter; polymyxin B, 2.5 IU/liter, amphotericin B, 2.5 mg/liter) antibiotics (all from Sigma), for mouse and monkey experiments, respectively. *H. pylori* liquid cultures for mouse and monkey inoculation were grown in brucella broth with 5% NCS and antibiotic supplementation for approximately 24 h (optical density at 600 nm 0.35 to 0.45), pelleted by centrifugation, and suspended in brucella broth. All *H. pylori* cultures were grown at 37°C under microaerophilic conditions generated either by a 5% CO2 incubator or by a fixed 5% O_2_ concentration (Anoxomat, Advanced Instruments, Norwood, MA). A complete list of strains and plasmids is shown in Table S2.

### Animals and experimental challenge

Male and female specific pathogen free rhesus macaques aged 3 to 6 years were derived at the California National Primate Research Center from the day of birth using methods previously described to ensure that they had normal gastric histology and were free of *H. pylori* infection [57]. Animals were housed individually and fed commercial primate chow (Purina) and fruit, with water available *ad libitum*. Macaques were orogastrically inoculated by endoscopy with 10^9^ CFU of *H. pylori* suspended in 2 ml of brucella broth. Endoscopy with gastric biopsy was performed with ketamine anesthesia (10 mg/kg given intramuscularly) after an overnight fast at defined time points PI. Specific-pathogen (*Helicobacter*)-free, female C57BL/6 and RAG2−/− mice (Taconic, Germantown, NY), or C57BL/6 and RAG1−/− mice (Jackson Laboratories) were housed in microisolator cages and provided with irradiated food and autoclaved water *ad libitum*. At 10 to 12 weeks of age mice were fasted for 3 to 4 hr and then challenged with 2.5×10^9^ CFU of *H. pylori* suspended in 0.25 ml of brucella broth administered by oral gavage with a ball-end feeding needle. All mice were euthanized between 2 and 16 weeks post inoculation (PI) with an overdose of pentobarbital sodium injection (50 mg/ml IP). Stomachs were cut longitudinally, and half was placed in brucella broth, weighed, ground with a sterile glass rod until the mucosal cells were homogenized, and then plated quantitatively by serial dilution on brucella agar supplemented with 5% NCS and ABPNV. Multiple single colony isolates recovered from mice and monkeys were characterized for their capacity to induce IL-8 and translocate CagA. All animals were housed under protocols approved by ALAAC and the U.C. Davis Institutional Animal Care and Use Committee.

### IL-8 ELISA

IL-8 was measured essentially as described previously [58]. Approximately 2.5×10^5^ human AGS gastric adenocarcinoma cells (ATCC, Manassas, VA) were seeded in six well plates, washed two times with 1× PBS, and overlaid with 1.8 ml RPMI/10% fetal bovine serum and bacteria diluted in 200 µl brucella broth to give an MOI of 100∶1. Brucella broth with no bacteria served as a baseline control. Supernatants were harvested after 22 hours of culture (37°C, 5% CO_2_), stored at −80°C, and then diluted 1∶4 prior to IL-8 assay by ELISA (Invitrogen, Camarillo, CA) performed according to the manufacturer's protocol. WT *H. pylori* J166 and its isogenic *cagY* deletion were included on every plate as positive and negative controls, respectively. Results in AGS cells were confirmed selectively using KATO III gastric adenocarcinoma cells (ATCC, Manassas, VA) grown in RPMI 1640 (Gibco BRL, Grand Island, NY) with 20% fetal bovine serum. To account for variability in the assay, IL-8 values were normalized to WT *H. pylori* determined concurrently.

### NF-κB reporter assay

AGS cells stably transfected with an NF-κB luciferase reporter (Promega E849A, Madison, WI) were plated without antibiotics in a 48-well plate at a density of 3×10^4^ cells per well for 24 hr prior to co-culture. *H. pylori* strains were grown overnight in liquid culture, diluted 10-fold in fresh media, and re-incubated for 4 hr to achieve log phase growth. Bacterial cells were washed once in sterile PBS and co-cultured with the AGS cells at an MOI of 10∶1 for 4 hr. Phorbol myristate acetate (PMA, 0.5 µg/mL) was used as a positive control. After 4 hr of co-culture, the media was removed, 100 µL/well of lysis buffer (Promega E4030) was added and mixed on an orbital shaker at 500 rpm for 10 min. To measure the luciferase activity, 100 µL of substrate (Promega E4030) and 20 µL of cell lysate were mixed and immediately read in a luminometer.

### Immunoblots and CagA translocation

Expression of CagA, phosphorylated CagA, and CagY were detected by immunoblot. For detection of CagA translocation, AGS cells were washed twice with 2 ml RPMI 1640 (Invitrogen) containing 1 mM sodium orthovanadate, and pelleted by centrifugation (14,000 g, 30 sec). Pellets were lysed in 100 µl of NENT (1% NP40, 5 mM EDTA, 250 mM NaCl, 25 mM Tris, 1 mM sodium orthovanadate, 1 mM phenylmethylsulfonyl fluoride), centrifuged (14,000 g, 3 min), and electrophoresed in a 7.5% polyacrylamide gel (BioRad, Hercules, CA). Proteins were transferred to a PVDF membrane (Millipore, Billerica, MA), blocked overnight in 3% BSA in TTBS (20 mM Tris-HCl, pH 7.5, 150 mM NaCl, 0.05% Tween 20, 3% bovine serum albumin), and incubated for 1 hr with mouse anti-phosphotyrosine IgG (Santa Cruz Biotechnology, Santa Cruz, CA) diluted 1∶5,000. Blots were washed three times for 5 min each in TTBS and incubated for 1 hr with horseradish peroxidase (HRP)-conjugated anti-mouse IgG (GE Healthcare, Buckinghamshire, UK) diluted 1∶10,000. Bound antibody was detected with chemiluminescence using ECL reagents (GE Healthcare, Bukinghamshire, UK). The blot was then incubated in stripping buffer (0.1 M β-mercaptoethanol, 10% SDS and 0.5 M Tris, pH 6.8) for 30 min at 50°C, washed and blocked as before, and immunoblotted for 1 hr with rabbit IgG antibody (1∶5,000) to CagA (Austral Biological, San Ramon, CA). Blots were washed in TTBS, incubated for 1 hr with anti-rabbit HRP-conjugated IgG (GE Healthcare, Buckinghamshire, UK) at 1∶20,000 dilution, and visualized by chemiluminescence. CagY expression was detected by electrophoresis of sonicated bacterial proteins on a 7.5% polyacrylamide gel, incubating with rabbit antiserum (1∶10,000) to CagY [18] as primary antibody and HRP-conjugated anti-rabbit IgG (1∶20,000) as secondary antibody, followed by chemiluminescent detection.

### *cagY* PCR-RFLP

*cagY* genotyping was performed by polymerase chain reaction-restriction fragment length polymorphism (PCR-RFLP). A fragment containing the *cagY* gene was PCR amplified using the Expand Long Template PCR System (Roche Diagnostics, Indianapolis, IN). Reactions were performed in a total volume of 50-µl containing 100 ng of genomic DNA, 0.3 µM of each primer (sense 5′-CCGTTCATGTTCCATACATCTTTG-3′; anti-sense 5′-CTATGGTGAATTGGAGCGTGTG -3′), 0.35 mM of each dNTP, 3.75 U of Expand *Taq* DNA polymerase, and 1× buffer containing 1.75 mM MgCl_2_. PCR products were purified (QIAquick PCR Purification Kit, QIAGEN Sciences, Maryland, MD), adjusted to a concentration of 120 µg/ml, and digested overnight at 37°C separately with DdeI, BfucI, and HinfI (New England BioLabs, Ipswich, MA). Digested DNA was separated by 3% (HinfI) or 5% (DdeI, BfucI) agarose gel electrophoresis and then stained with ethidium bromide. Gels were examined and *cagY* from each output colony was determined to be the same as that of the J166 input strain if RFLP patterns were identical for all three restriction enzymes. Oligonucleotide primers for amplification, sequencing, and PCR-RFLP analysis of *cagY* are shown in Table S3.

### Contraselection for genetic exchange of *cagY*

Alleles of *cagY* were exchanged between *H. pylori* strains using contraselectable streptomycin susceptibility [26] modified essentially as described previously [27]. The 1,420 bp *cat-rpsL* cassette encoding chloramphenicol resistance and dominant streptomycin susceptibility was amplified with primers (RpsLF, C2CamR) that contained SacI and BamHI restriction sites, ligated between fragments of DNA upstream (1,348 bp, primers *cagX*F, *cagY*R) and downstream (1,122 bp, primers *cagY*F and virB11R) of *cagY* that contained complementary restriction sites, and cloned into pBluescript (Stratagene, La Jolla, CA). *H. pylori* was made streptomycin resistant by transformation with genomic DNA from a mutant of strain 26695, which contained an A-to-G change at codon 43 of *rpsL*, and selection on streptomycin (10 µg/ml). Transformation of streptomycin-resistant *H. pylori* with plasmid containing the *cat-rpsL* cassette and flanking *cagY* sequences, with selection on chloramphenicol (5 µg/ml), resulted in the replacement of bp 13 to 6,135 of *cagY*. The *cagY* gene of interest was then reinserted by transformation of the *cagY* knockout with genomic DNA from the donor strain and selection on streptomycin. Streptomycin-resistant, chloramphenicol-sensitive colonies were fully sequenced at the *cagY* locus to confirm that they had undergone the desired genetic exchange.

### FEG-SEM of T4SS pili

*H. pylori* was imaged by FEG-SEM using methods previously described [15]. In brief, *H. pylori* and AGS human gastric cells were co-cultured at an MOI of 100∶1 on tissue culture-treated coverslips (BD Biosciences) for 4 h at 37°C in the presence of 5% CO2. Cells were fixed with 2.0% paraformaldehyde, 2.5% glutaraldehyde in 0.05 M sodium cacodylate buffer for 1 hr at 37°C. Coverslips were washed with sodium cacodylate buffer and secondary fixation was performed with 1% osmium tetroxide at room temperature for 30 min. Coverslips were washed with sodium cacodylate buffer and dehydrated with sequential washes of increasing concentrations of ethanol. Samples were then dried at the critical point, mounted onto sample stubs, grounded with a thin strip of silver paint at the sample edge, and sputter-coated with palladium-gold before viewing with an FEI Q250 FEG scanning electron microscope. Image analysis was performed using Image J software.

### Immunogold SEM

Bacteria were co-cultured with AGS cells and fixed as for FEG-SEM. Cells were then washed three times in 0.05 M sodium cacodylate buffer before blocking in 0.1% cold fish skin gelatin in 0.05 M sodium cacodylate buffer for 1 hr. Primary polyclonal rabbit antibodies to CagA and the CagY MRR [18] were applied overnight followed by three buffer washes and application of secondary goat anti-rabbit antibody conjugated to 20 nm gold particle (Electron Microscopy Sciences, Hatfield, PA) for 4 hr. After three buffer washes, cells were fixed again (2.0% paraformaldehyde, 2.5% glutaraldehyde in 0.05 M sodium cacodylate) for 1 hr to stabilize the antibody interactions, washed, and then treated with 0.1% osmium tetroxide for 15 min followed by three additional buffer washes and sequential ethanol dehydration. Cells were dried at the critical point and carbon-coated before imaging with an FEI Quanta 250 FEG-SEM. Gold particles were confirmed with backscatter imaging analysis. As negative controls, uninfected AGS cells were processed in parallel, or application of the primary antibody was omitted.

### DNA sequencing

*cag*PAI genes known to be involved in IL-8 induction were amplified and sequenced using primers shown in Table S3. *cagY* genes were amplified with primers in flanking genes using Expand Long Template PCR system (Roche, Indianapolis, IN). Purified PCR products were cloned into pDrive (Qiagen, Valencia, CA) and plasmids were sequenced with dye terminator chemistry. PCR products were sometimes sequenced directly for verification. To confirm the number of 390 bp repeats in the FRR, the *cagY* PCR products were run on 0.4% agarose gels at 18 volts for 16 hr. The size of the PCR product minus 477 bp gave an estimate of total *cagY* size. All DNA sequences of *cagY* have been deposited in GenBank under accession numbers JQ685133–JQ685155.

### Statistical analysis

Data were analyzed using a 2-tailed Student's *t* test (Prism 5.0) unless otherwise indicated. A *P* value<0.05 was considered statistically significant.

## Supporting Information

Figure S1 ***H. pylori*** **induction of IL-8 is similar in AGS and KATO III gastric adenocarcinoma cells lines, related to** **Figures 1** **–** **4** **.** Normalized induction of IL-8 in AGS cells (filled bars) and KATO III cells (hatched bars) after co-culture with WT *H. pylori*, its *cagY* deletion mutant (▵Y), and output strains recovered from monkeys (A) and mice (B) that induce low (Out1, Out2) or high (Out3, Out4) IL-8. Results are normalized to WT and expressed as the mean ± SEM of 3 replicates. **P<0.01; ***P<0.001.
(TIF)

Figure S2 ***H. pylori*** **J166** ***cagY*** **has a large number of direct DNA repeats that are organized into a 5′ repeat region (FRR) and a middle repeat region (MRR).** JDotter (http://athena.bioc.uvic.ca/tools/JDotter) was used to generate a dot plot comparing the 6,171 bp *cagY* gene in *H. pylori* J166 to itself. Each position at which the base pairs are identical is marked with a dot. Sequence identity of the two genes generates a single diagonal line from 0 to 6,171 bp. Direct DNA repeats in the FRR and MRR are indicated by shorter lines that are symmetrical about the diagonal. The *cagY* gene in *H. pylori* strains J99 and 26695 is organized similarly [18].
(TIF)

Figure S3 ***H. pylori*** **strains bearing variant** ***cagY*** **alleles are selected during experimental infection, related to** **Figures 1** **–** **4** **.** Representative output strains recovered from monkeys (A) and C57BL/6 mice (B) were identified that induced low (Out1, Out2) or high (Out3, Out4) IL-8. *cagY* from WT *H. pylori* J166 (input) and each output strain was amplified from genomic DNA, digested individually with DdeI, Hinf1, and Bfuc1 (an isoschizomer of Sau3AI), and examined by 3% (Hinf1) or 5% (DdeI and Bfuc1) agarose gel electrophoresis. Each strain showed a unique fingerprint except rOut1 and mOut1, which were demonstrated to be identical by DNA sequence analysis. Size ladder in base pairs (bp) is shown next to each gel. PCR-RFLP patterns from 85 output strains from mice and monkeys were judged by three independent observers, who demonstrated 100% agreement.
(TIF)

Figure S4 **CagA is required for full induction of IL-8 in** ***H. pylori*** **J166, related to** **Figure 1** **.** Deletion of *cagA* (▵A) in *H. pylori* J166 significantly reduced its capacity to induce IL-8 (mean ± SEM of 3 replicates) compared to WT, though IL-8 remained higher than in a strain with deletion of *cagY* (▵Y). ***P<0.001.
(TIF)

Figure S5 ***H. pylori*** **colonization of WT C57BL/6 mice and RAG2−/− mice that do not have functional B or T cells, related to** **Figures 3** **and** **10B** **.** Colonization density in WT C57BL/6 mice was significantly lower than in RAG2−/− mice infected with WT *H. pylori* J166 (A) or with mouse output strain mOut2 (B). Results are shown as mean ± SEM log10 CFU/g up to 16 weeks PI. *P<0.05; **P<0.01; ***P<0.001.
(TIF)

Figure S6 **Complementation of mOut2 with WT** ***cagY*** **restores its capacity to induce IL-8, related to** **Figure 10** **.** Complementation of *cagY* in mOut2 with that from WT *H. pylori* J166 restored its capacity to induce IL-8 to that of WT J166. All assays represent the mean ±SEM of 3 replicates. ***P<0.001.
(TIF)

Figure S7 **Persistence in one monkey of a variant** ***cagY*** **strain that induces IL-8, related to** **Figure 10** **.** Monkey 36001 was inoculated with mOut2, which has a variant *cagY* allele and does not induce IL-8 or phosphorylate CagA. Repeated sampling of monkey 36001 up to 24 wks PI showed that all output colonies recovered 8 wks or more PI induced IL-8 and expressed a *cagY* that differed from that in mOut2.
(TIF)

Table S1 **Quantitative analysis of** ***H. pylori*** **pili by FEG-SEM.**
(DOC)

Table S2 **Bacterial strains and plasmids.**
(DOC)

Table S3 **DNA primers used for PCR (bold) and sequencing.**
(DOC)
