# Supplementary figures and images for: Functional Plasticity in the Type IV Secretion System of Helicobacter pylori
Source: PLoS Pathog. 2013 Feb 28;9(2):e1003189. doi: 10.1371/journal.ppat.1003189 (PMC3585145; doi:10.1371/journal.ppat.1003189)

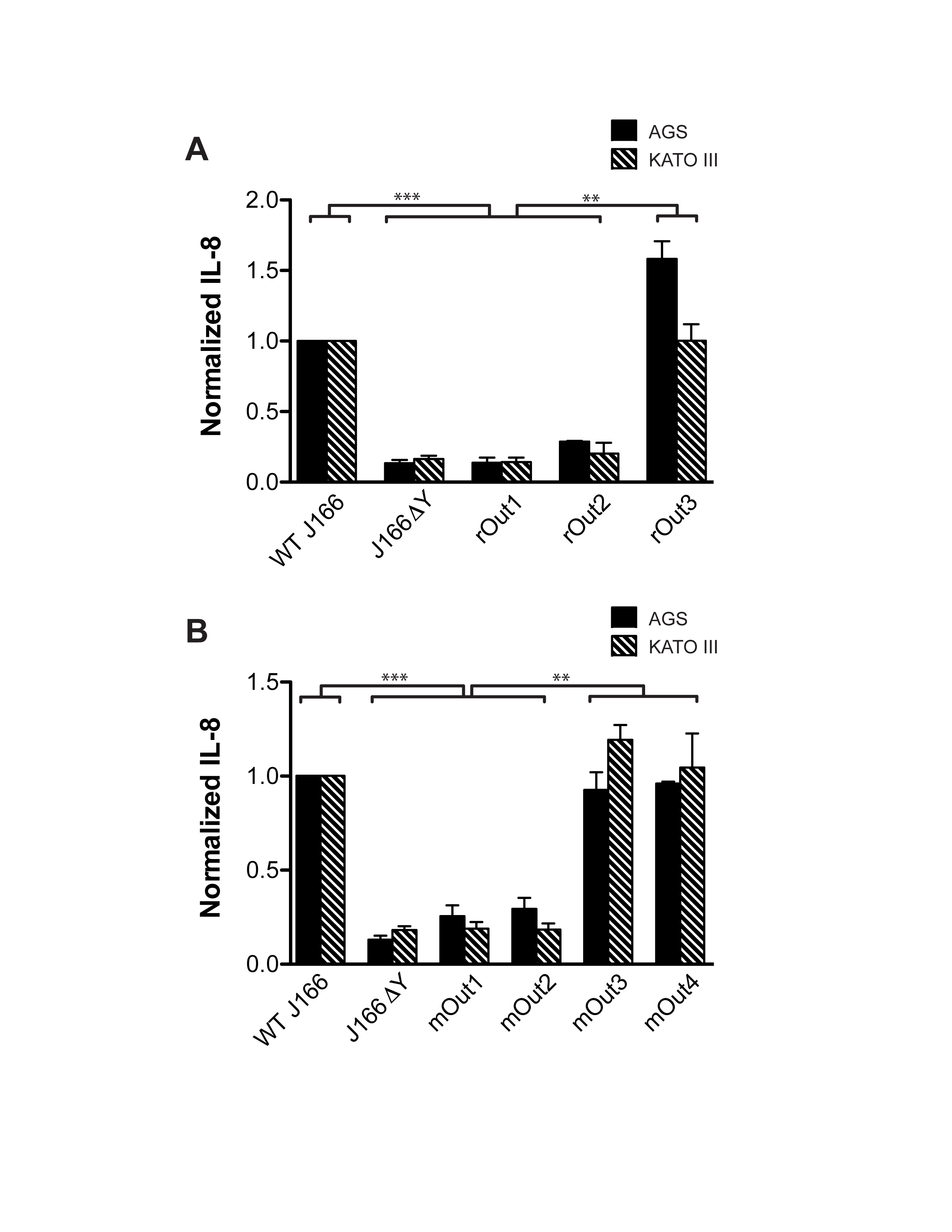

Supplement: Figure S1 — H. pylori induction of IL-8 is similar in AGS and KATO III gastric adenocarcinoma cells lines, related to Figures 1 – 4 . Normalized induction of IL-8 in AGS cells (filled bars) and KATO III cells (hatched bars) after co-culture with WT H. pylori, its cagY deletion mutant (▵Y), and output strains recovered from monkeys (A) and mice (B) that induce low (Out1, Out2) or high (Out3, Out4) IL-8. Results are normalized to WT and expressed as the mean ± SEM of 3 replicates. **P<0.01; ***P<0.001. (TIF) [file ppat.1003189.s001.tif]

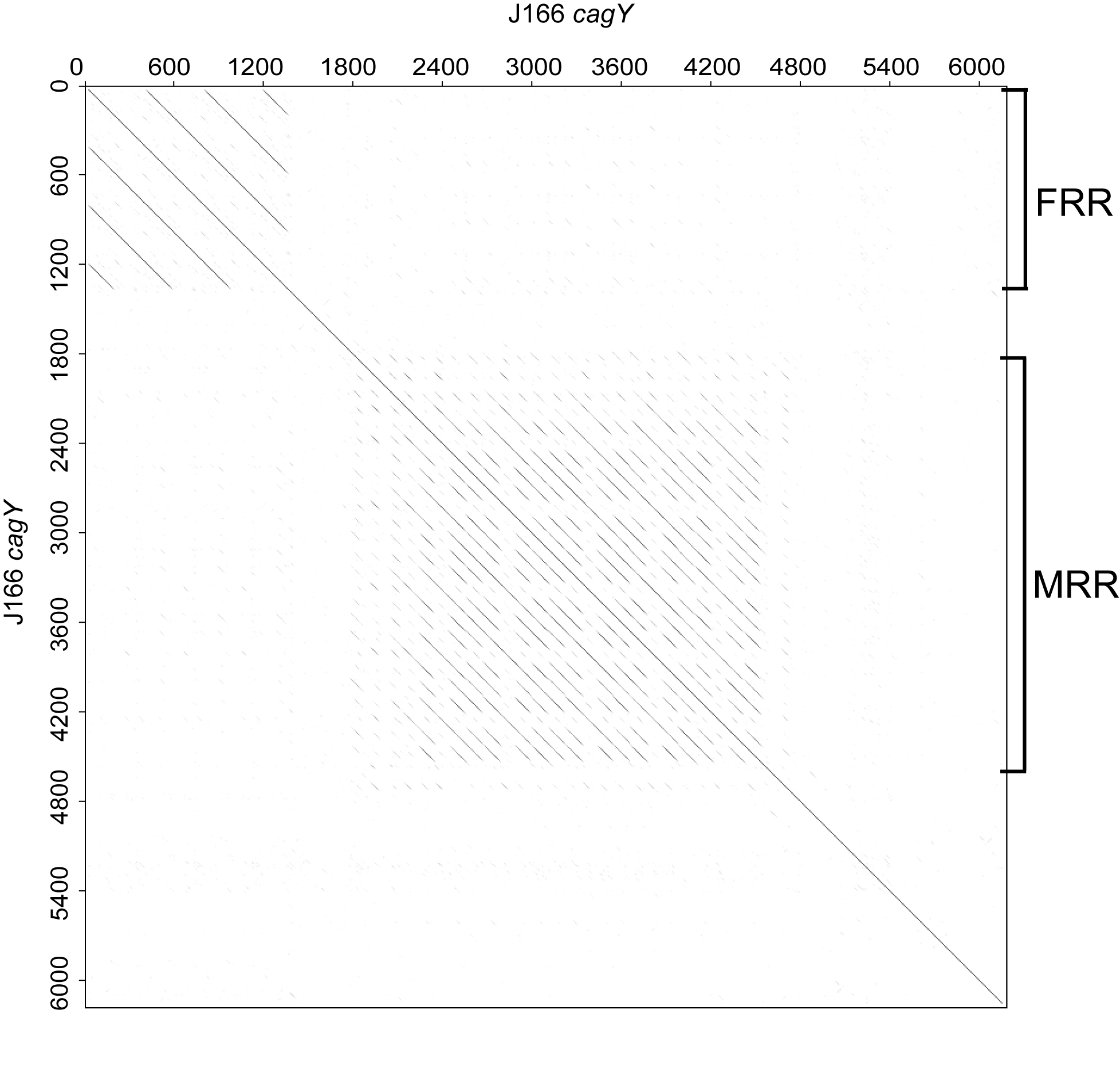

Supplement: Figure S2 — H. pylori J166 cagY has a large number of direct DNA repeats that are organized into a 5′ repeat region (FRR) and a middle repeat region (MRR). JDotter (http://athena.bioc.uvic.ca/tools/JDotter) was used to generate a dot plot comparing the 6,171 bp cagY gene in H. pylori J166 to itself. Each position at which the base pairs are identical is marked with a dot. Sequence identity of the two genes generates a single diagonal line from 0 to 6,171 bp. Direct DNA repeats in the FRR and MRR are indicated by shorter lines that are symmetrical about the diagonal. The cagY gene in H. pylori strains J99 and 26695 is organized similarly [18]. (TIF) [file ppat.1003189.s002.tif]

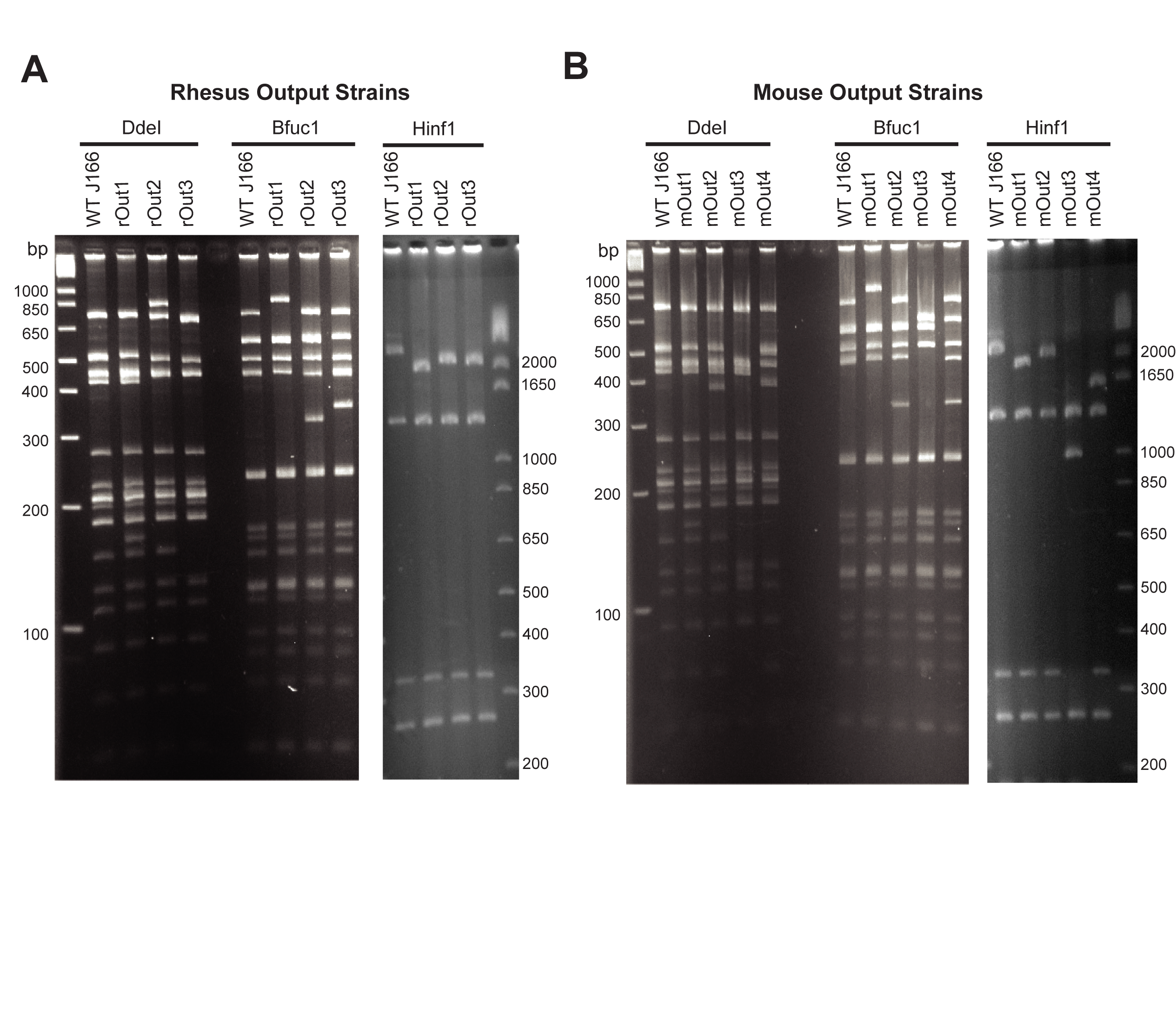

Supplement: Figure S3 — H. pylori strains bearing variant cagY alleles are selected during experimental infection, related to Figures 1 – 4 . Representative output strains recovered from monkeys (A) and C57BL/6 mice (B) were identified that induced low (Out1, Out2) or high (Out3, Out4) IL-8. cagY from WT H. pylori J166 (input) and each output strain was amplified from genomic DNA, digested individually with DdeI, Hinf1, and Bfuc1 (an isoschizomer of Sau3AI), and examined by 3% (Hinf1) or 5% (DdeI and Bfuc1) agarose gel electrophoresis. Each strain showed a unique fingerprint except rOut1 and mOut1, which were demonstrated to be identical by DNA sequence analysis. Size ladder in base pairs (bp) is shown next to each gel. PCR-RFLP patterns from 85 output strains from mice and monkeys were judged by three independent observers, who demonstrated 100% agreement. (TIF) [file ppat.1003189.s003.tif]

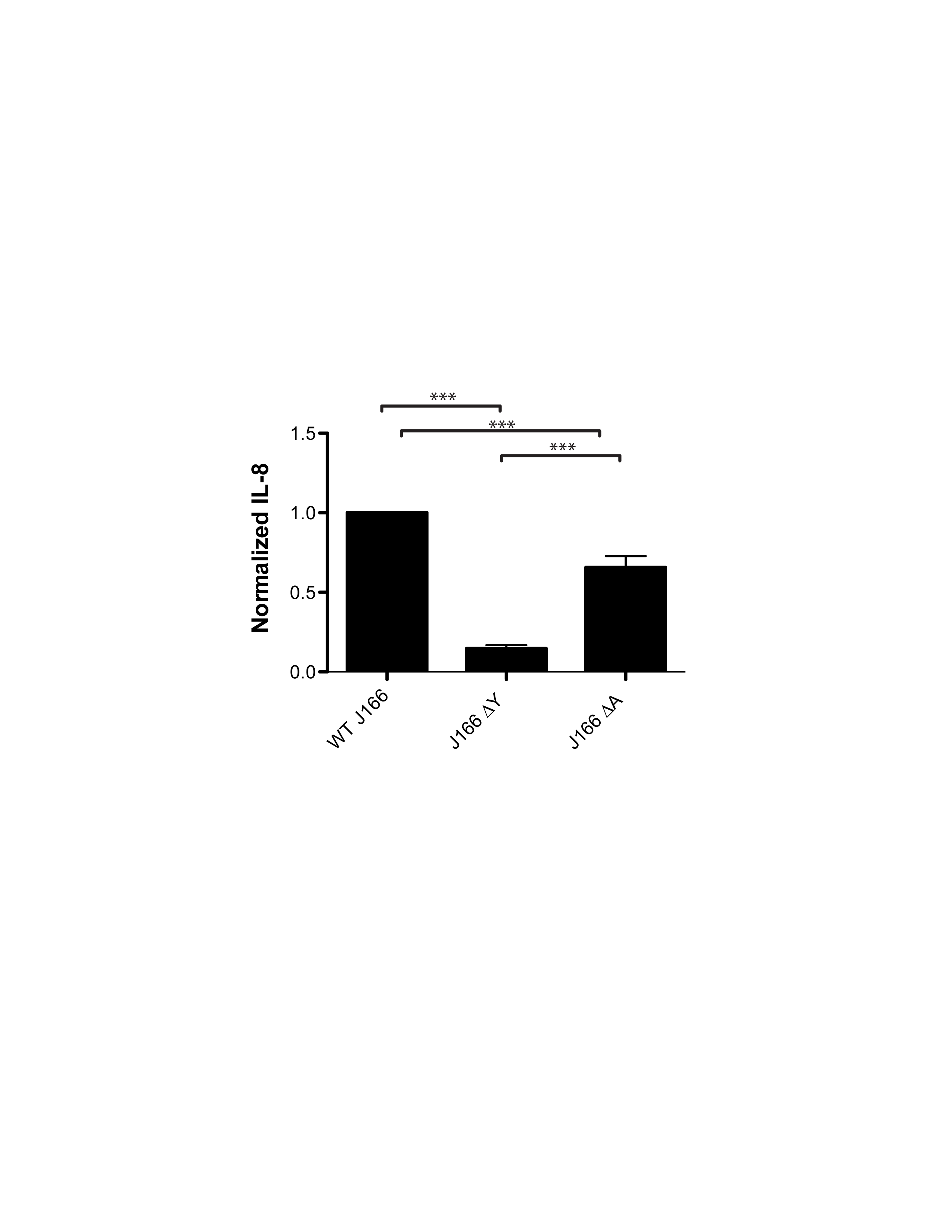

Supplement: Figure S4 — CagA is required for full induction of IL-8 in H. pylori J166, related to Figure 1 . Deletion of cagA (▵A) in H. pylori J166 significantly reduced its capacity to induce IL-8 (mean ± SEM of 3 replicates) compared to WT, though IL-8 remained higher than in a strain with deletion of cagY (▵Y). ***P<0.001. (TIF) [file ppat.1003189.s004.tif]

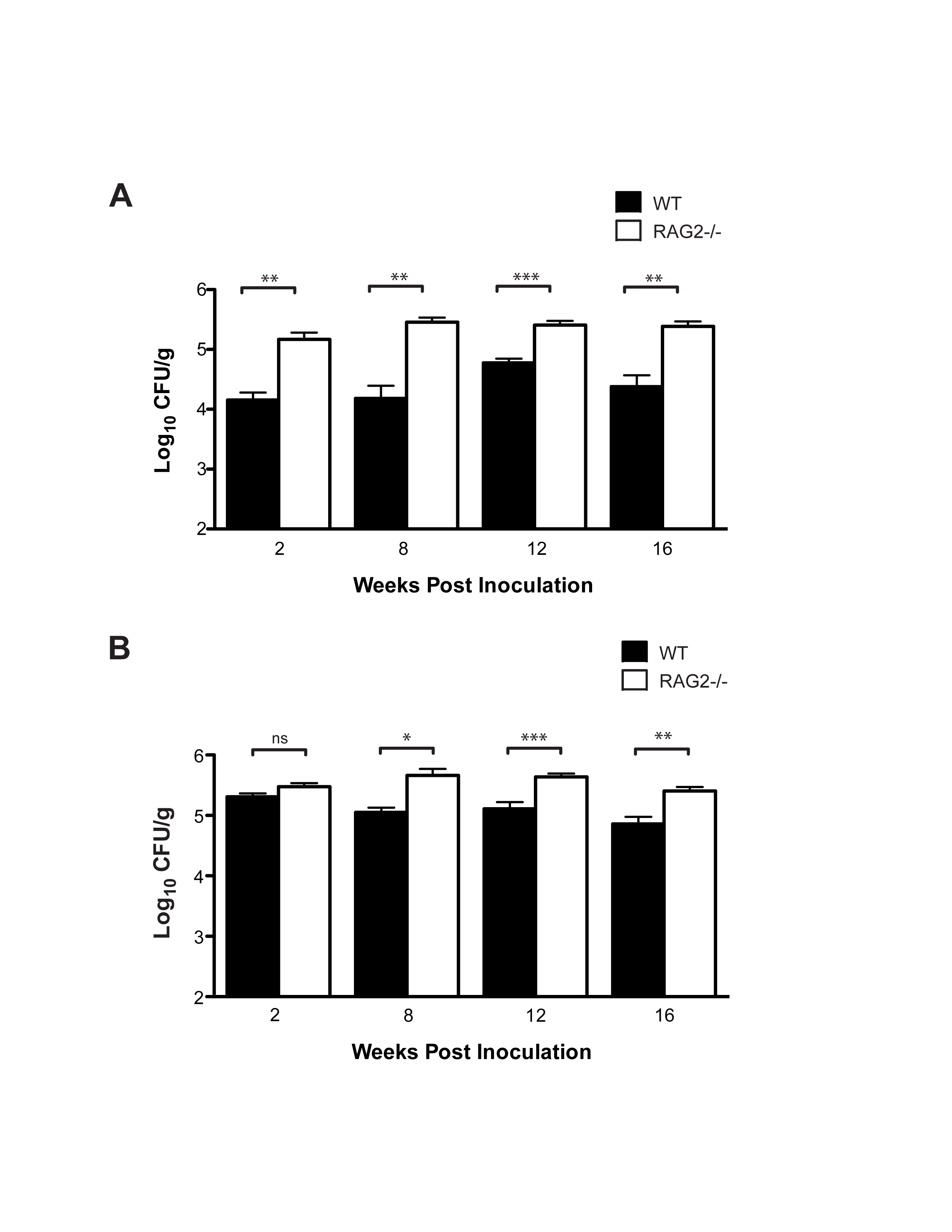

Supplement: Figure S5 — H. pylori colonization of WT C57BL/6 mice and RAG2−/− mice that do not have functional B or T cells, related to Figures 3 and 10B . Colonization density in WT C57BL/6 mice was significantly lower than in RAG2−/− mice infected with WT H. pylori J166 (A) or with mouse output strain mOut2 (B). Results are shown as mean ± SEM log10 CFU/g up to 16 weeks PI. *P<0.05; **P<0.01; ***P<0.001. (TIF) [file ppat.1003189.s005.tif]

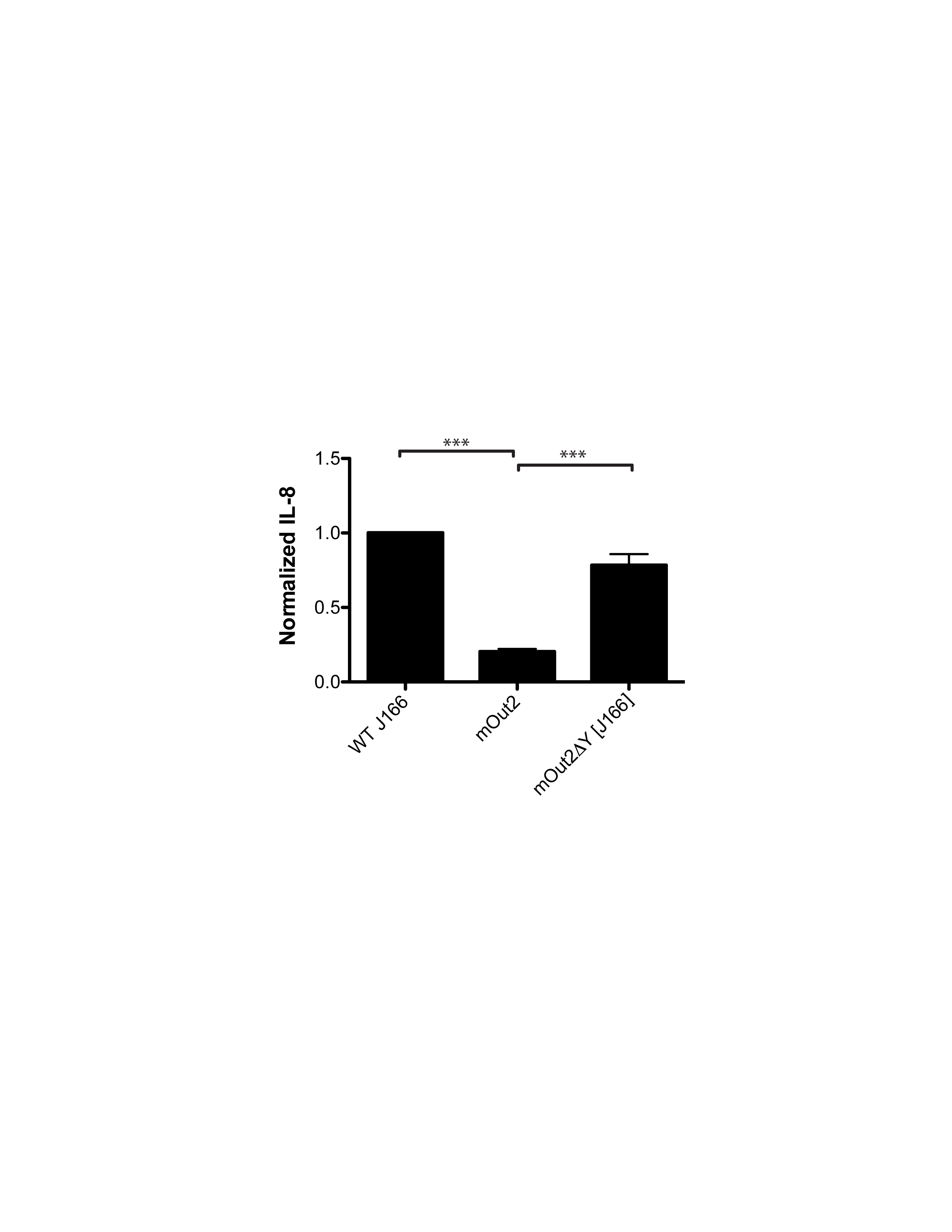

Supplement: Figure S6 — Complementation of mOut2 with WT cagY restores its capacity to induce IL-8, related to Figure 10 . Complementation of cagY in mOut2 with that from WT H. pylori J166 restored its capacity to induce IL-8 to that of WT J166. All assays represent the mean ±SEM of 3 replicates. ***P<0.001. (TIF) [file ppat.1003189.s006.tif]

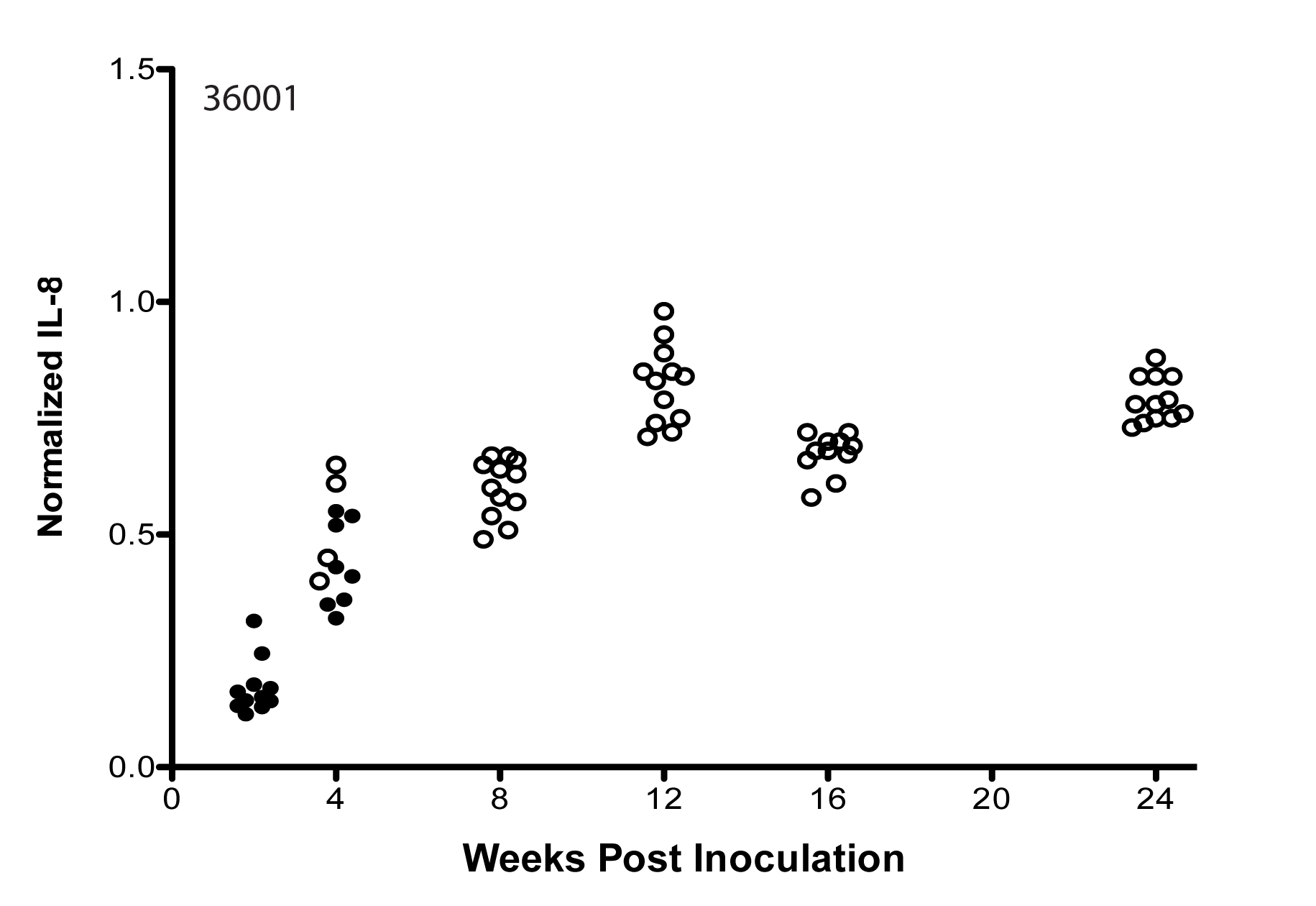

Supplement: Figure S7 — Persistence in one monkey of a variant cagY strain that induces IL-8, related to Figure 10 . Monkey 36001 was inoculated with mOut2, which has a variant cagY allele and does not induce IL-8 or phosphorylate CagA. Repeated sampling of monkey 36001 up to 24 wks PI showed that all output colonies recovered 8 wks or more PI induced IL-8 and expressed a cagY that differed from that in mOut2. (TIF) [file ppat.1003189.s007.tif]
