# Supplementary material for: Functional Plasticity in the Type IV Secretion System of Helicobacter pylori
Source: PLoS Pathog. 2013 Feb 28;9(2):e1003189. doi: 10.1371/journal.ppat.1003189 (PMC3585145; doi:10.1371/journal.ppat.1003189)
Supplement: Table S1 — Quantitative analysis of H. pylori pili by FEG-SEM. (DOC) [file ppat.1003189.s008.doc]

| Supplementary Table 1. Quantitative analysis of *H. pylori* pili (mean ± SEM)^a^ | | | | | | |
| --- | --- | --- | --- | --- | --- | --- |
| *H. pylori* strain | IL-8 | Pili/cell | Pili/µm/cell | Pili width (nm) | Pili length (nm) | Space between pili |
| J166 WT | 1.00 (.00) | 13.9 (1.4) | 13.9 (1.4) | 12.1 (0.4) | 92.8 (4.2) | 61.9 (5.9) |
| J166△*cagY*:WT *cagY* | .85 (.02) | 10.7 (2.9) | 15.5 (1.7) | 11.7 (0.4) | 92.8 (5.0) | 61.2 (6.1) |
| J166△*cagY*:rOut3 *cagY* | 1.67 (.15) | 23.1 (2.8)* | 18.7 (1.3) | 10.8±0.7 | 146.4 (10.0)** | 72.9 (6.6) |
| J166△*cagY*:mOut3 *cagY* | .97 (.06) | 14.2 (1.8) | 17.2 (0.9) | 11.4±0.4 | 87.9 (7.0) | 64.0 (6.0) |
| J166△*cagY*:mOut4 cagY | .88 (.04) | 6.2 (2.4)** | 3.5 (1.1)** | 11.9±0.4 | 103.1 (11.3) | 338.9 (52.3)** |
|  |  |  |  |  |  |  |
| J166△*cagY* | .29 (.04) | 31.4 (6.0) | 19.6 (1.5) | 11.8 (0.3) | 38.3 (2.8)** | 43.2 (2.8)* |
| J166△PAI | .22 (.01) | 0.0 (0.0) | 0.0 (0.0)** | ND | ND | ND |
| J166△*cagY*:rOut1 *cagY* | .20 (.03) | 5.0 (1.1)** | 7.7 (0.9)* | 12.0 (0.2) | 103.2 (10.6) | 191.3 (41.1)* |
| J166△*cagY*:rOut2 *cagY* | .39 (.05) | 18.4 (3.4) | 15.1 (1.7) | 12.7 (0.7) | 53.0 (3.2)** | 81.3 (12.6) |
| J166△*cagY*:mOut1 *cagY* | .21 (.03) | 4.9 (0.8)** | 5.1 (0.9)** | 10.8 (0.3) | 56.6 (3.8)** | 164.4 (29.7)* |
| J166△*cagY*:mOut2 *cagY* | .23 (.03) | 11.9 (1.7)* | 14.0 (1.8) | 11.8 (0.3) | 55.6 (4.2)** | 135.4 (29.9) |

^a^Based on visualization of ≥ 3 biological replicates and ≥ 40 total adherent cells.

**P*<.01, ***P*<.001 compared to J166 WT by 2-tailed T-test; ND=no detectable pili
