# Supplementary material for: Functional Plasticity in the Type IV Secretion System of Helicobacter pylori
Source: PLoS Pathog. 2013 Feb 28;9(2):e1003189. doi: 10.1371/journal.ppat.1003189 (PMC3585145; doi:10.1371/journal.ppat.1003189)
Supplement: Table S2 — Bacterial strains and plasmids. (DOC) [file ppat.1003189.s009.doc]

| **Supplementary Table 2. Strains and plasmids** | | |  |  |
| --- | --- | --- | --- | --- |
| **Strain or plasmid** |  | **Relevant characteristic(s)** | **Antibiotic resistance** | **Reference or source** |
| Strains |  |  |  |  |
| *E. coli* |  |  |  |  |
| Top 10 |  | Cloning strain |  | Invitrogen |
| *H. pylori* |  |  |  |  |
| J166 |  | Wild type |  | [56] |
| PMSS1 |  | Wild type |  | [35] |
| SS1 |  | mouse passaged PMSS1 |  | [32] |
| 26695 Str^R^ |  | mutation at codon 43 of *rpsL* (K43R) | Str | [25] |
| J166 Str^R^ |  | mutation at codon 43 of *rpsL* gene (K43R) | Str | this study |
| J166 Δ*cag*PAI |  | Deletion of the entire *cag*PAI | Cm, Km | [42] |
| Rhesus passaged |  |  |  |  |
| rOut1 |  | J166 from rhesus gastric antrum 14 months post inoculation |  | [22] |
| rOut2 |  | J166 from rhesus gastric antrum 14 months post inoculation |  | [22] |
| rOut3 |  | J166 from rhesus gastric antrum 4 months post inoculation |  | [22] |
| Mouse passaged |  |  |  |  |
| mOut1 |  | J166 from mouse stomach 16 weeks post inoculation |  | this study |
| mOut2 |  | J166 from mouse stomach 16 weeks post inoculation |  | this study |
| mOut2 Str^R^ |  | mutation at codon 43 of *rpsL* gene (K43R) | Str | this study |
| mOut3 |  | J166 from mouse stomach 16 weeks post inoculation |  | this study |
| mOut4 |  | J166 from mouse stomach 16 weeks post inoculation |  | this study |
| *cagY* deletions |  |  |  |  |
| J166Δ*cagY* |  | J166 Str^R^ Δ*cagY*::*cat_rpsL* | Cm | this study |
| mOut2Δ*cagY* |  | mOut2 Str^R^ Δ*cagY*::*cat_rpsL* | Cm | this study |
| *cagY* replacements | | |  |  |
| Δ*cagY*[rOut1] |  | J166Δ*cagY* replaced with *cagY* from rOut1 | Str | this study |
| Δ*cagY*[rOut2] |  | J166Δ*cagY* replaced with *cagY* from rOut2 | Str | this study |
| Δ*cagY*[rOut3] |  | J166Δ*cagY* replaced with *cagY* from rOut3 | Str | this study |
| Δ*cagY*[mOut1] |  | J166Δ*cagY* replaced with *cagY* from mOut1 | Str | this study |
| Δ*cagY*[mOut2] |  | J166Δ*cagY* replaced with *cagY* from mOut2 | Str | this study |
| Δ*cagY*[mOut3] |  | J166Δ*cagY* replaced with *cagY* from mOut3 | Str | this study |
| Δ*cagY*[mOut4] |  | J166Δ*cagY* replaced with *cagY* from mOut4 | Str | this study |
| Δ*cagY*[SS1] |  | J166Δ*cagY* replaced with *cagY* from SS1 | Str | this study |
| Δ*cagY*[PMSS1] |  | J166Δ*cagY* replaced with *cagY* from PMSS1 | Str | this study |
| mOut2Δ*cagY*[J166] |  | mOut2Δ*cagY* replaced with *cagY* from J166 WT | Str | this study |
| Plasmids |  |  |  |  |
| pBluescript SK- |  | Cloning vector | Ap | Stratagene |
| pDrive |  | Cloning vector | Ap, Km | Qiagen |
| pJ261 |  | pBluescript SK- with J166 *cagY* and flanking region inserted*; (cagY* bp 13-6135 replaced by *CAT_rpsL)* | Ap, Cm | this study |
| pJ294 |  | pDrive with J166 *cagY* and flanking region inserted | Ap, Km | this study |
| pJ295 |  | pDrive with rOut1 *cagY* and flanking region inserted | Ap, Km | this study |
| pJ296 |  | pDrive with Δ*cagY*[rOut1] *cagY* and flanking region inserted | Ap, Km | this study |
| pJ304 |  | pDrive with rOut2 *cagY* and flanking region inserted | Ap, Km | this study |
| pJ305 |  | pDrive with Δ*cagY*[rOut2] *cagY* and flanking region inserted | Ap, Km | this study |
| pJ298 |  | pDrive with rOut3 *cagY* and flanking region inserted | Ap, Km | this study |
| pJ299 |  | pDrive with Δ*cagY*[rOut3] *cagY* and flanking region inserted | Ap, Km | this study |
| pJ281 |  | pDrive with mOut1 *cagY* and flanking region inserted | Ap, Km | this study |
| pJ279 |  | pDrive with Δ*cagY*[mOut1] *cagY* and flanking region inserted | Ap, Km | this study |
| pJ292 |  | pDrive with mOut2 *cagY* and flanking region inserted | Ap, Km | this study |
| pJ287 |  | pDrive with Δ*cagY*[mOut2] *cagY* and flanking region inserted | Ap, Km | this study |
| pJ275 |  | pDrive with mOut3 *cagY* and flanking region inserted | Ap, Km | this study |
| pJ276 |  | pDrive with Δ*cagY*[mOut3] *cagY* and flanking region inserted | Ap, Km | this study |
| pJ252 |  | pDrive with mOut4 *cagY* and flanking region inserted | Ap, Km | this study |
| pJ290 |  | pDrive with Δ*cagY*[mOut4] *cagY* and flanking region inserted | Ap, Km | this study |
|  |  |  |  |  |
