# Supplementary material for: Functional Plasticity in the Type IV Secretion System of Helicobacter pylori
Source: PLoS Pathog. 2013 Feb 28;9(2):e1003189. doi: 10.1371/journal.ppat.1003189 (PMC3585145; doi:10.1371/journal.ppat.1003189)
Supplement: Table S3 — DNA primers used for PCR (bold) and sequencing. (DOC) [file ppat.1003189.s010.doc]

**Supplementary Table 3.** Primers used for PCR (bold) and sequencing

|  | **Name** | **Sequence (5' to 3')** |
| --- | --- | --- |
| Contraselection for genetic exchange of *cagY* | | |
|  | ***RpsL*F** | AAC GAG CTC GAT GCT TTA TAA CTA TGG ATT AAA CAC |
|  | **C2CamR** | AAC GGA TCC TTA TCA GTG CGA CAA ACT GGG AT |
|  | ***cagX*F** | AAC CTC GAG TAA AGG TTG GAG TAT TGT GCC TA |
|  | ***cagY*R** | AAC GAG CTC TTC TTC ATT CAT GTC TTA ACG C |
|  | ***cagY*F** | AAC GGA TCC CAT GAA GAA ATC ACC ACA AGC C |
|  | ***virB11*R** | AAC GCG GCC GCC ATT CGC TAA ATT GCT GCT CA |
| *cagY* amplification for PCR-RFLP | | |
|  | ***cagY*:5157L24** | CCG TTC ATG TTC CAT ACA TCT TTG |
|  | ***cagX*:1515U22** | CTA TGG TGA ATT GGA GCG TGT G |
| *cagY* amplification, cloning and sequencing | | |
|  | ***cagX*:1395U28** | ATG GTA TAG AGT TAA TGA AAT TGC AGA A |
|  | ***cagZ*:116L24** | CTT GCG GAT CGT TGC TAT CTT TTA |
|  | *cagY*:966U24 | GTC AGA AGA AAT AAC TAA CGA CTC |
|  | *cagY*:1525U20 | GTT CAA GTG GCG CTA GAT TG |
|  | *cagY*:2031U24 | AGA CTT ACA AAG CGA TAT TTT AGC |
|  | *cagY*:4235U21 | GTT TGA GCG ATG AAG AGA AGC |
|  | *cagY*:1043L24 | TCA CGA TAA GAA CAG CGA CTA CAA |
|  | *cagY*:1376L26 | CAT CTG ATC TTG AAG TTT TTT GTC TT |
|  | *cagY*:3803L28 | AGC ACT TGC TTC GCT AAG AAC TTT CTC G |
|  | *cagY*:4344L23 | CTT GGA TCA AAT CGC TAT AAA GG |
| *cag3,4* amplification and sequencing | | |
|  | **J166*cag1*_*3*:165U25** | GAA TTT GTC CAA TAG GGG ATT TTT A |
|  | ***cag4*_*5*:91L33** | AAA TAA AAA GAC AGA ATA TCT TAA CAA AAA GAA |
|  | *cag3*:377U22 | AAA CAA GAG CGA TGG GAA CTT A |
|  | *cag3*:690L19 | TAG GGG CGA ACA CAC TTC A |
|  | J99*cag4*:77L23 | GGT GAG ATT TTC GTA TTG CTT GA |
| *virB11* amplification and sequencing | | |
|  | ***cagZ*:483U26** | CAA TGA GAT GGT CCA AGA TAT AGG GA |
|  | ***cag5*:61L30** | AAA TAA CGC TAT TAA CCC TAT GAA ACT AAA |
| *cagW,X* amplification and sequencing | | |
|  | ***cagY*:2L26** | TTC AAG TTT ATC GTT TTC TTC ATT CA |
|  | ***cagV*:428U24** | TCG TAG ATA AAG TCC GAG CAG AAG |
|  | *cagW*:1534U25 | GAT GGT AGC AGA ATG GAT AGA GAA A |
|  | *cagW*:627L23 | CGA CAA AAG CAA GCA TGG CTG TA |
|  | *cagW*:1241L29 | CAT AGA ATC TTT GAA CCA ATC TAG AAC GA |
|  | *cagX*:959U21 | CTA GCG TTA TTG AAG AGG AGC |
|  | *cagX*:29L24 | AAT AAC CAA GAC AGA AAC AGC CAA |
|  | *cagX*:1033L27 | TCT ATT GAT GTA GGC TGT GGT GTT AAG |
|  | *cagX*:1545L21 | TTA TCT CTG ACA AGA GGG AGC |
| *cagT,U,V* amplification and sequencing | | |
|  | ***cagW*:53L26** | AAT AGC TTT CAA CCA ATT AGG AAC AA |
|  | ***cagS*:495U25** | CGC TAA TCT AAA AAC CAT TGA ACA A |
|  | *cagT*:162U23 | CAA ACT CAA AGA CAC ACC ATT CA |
|  | *cagU*:50U22 | CAC CTA GCA ACT CAC AGA GCA A |
|  | *cagV*:16U25 | AAC GAA GAA GTC TTG ATT GAT GAA A |
|  | *cagV*:198L23 | CTG TGT ATC GAT CAA TGC CAT AA |
| *cagL,M* amplification and sequencing | | |
|  | ***cagI*:1094U24** | AAA GAG AAA CGA CAG CAA GAA ACA |
|  | ***cagP*:219L30** | GAA ACG AGT AGC AAA AGA TAA GTA GTG TAA |
|  | *cagL*:110U22 | CCA ACC AAC AAG TGC TCA AAA A |
|  | *cagL*:512L25 | GTC TGT GAA GCA GTG ATT AAG GAA G |
|  | *cagM*:103U21 | GAA GAA GTG GCT GCA AAA GAA |
|  | *cagM*:530L24 | CTT CAT CTA AAG AAA ACA CAC CCA |
|  | *cagN*:217L23 | GCT CTT GCC CTA TCA TTT CGT AA |
| *cagI* amplification and sequencing | | |
|  | ***cagH*:805U23** | GCC GTT TAT TGC TAT GCT GAA AA |
|  | ***cagL*:223L25** | TCA ATA ACG CTA AAT CTC CTC TCA A |
|  | *cagI*:33U24 | GAC TTT TTG TGG TTT GTC TCT GAA |
| *cagH* amplification and sequencing | | |
|  | ***cagG*:341U19** | GCG ATT ACG GTC CAA ACA A |
|  | ***cagI*:306L20** | CGC CAA GCA AGA TGT CTG AA |
|  | *cagH*:1085U25 | GGG TGC AAA CTA AAA TAA TCG TGA A |
|  | *cagH*:690L25 | ATA AAC AGT GGG CAT AGA AAC ACT C |
|  | *cagI*:306L20 | CGC CAA GCA AGA TGT CTG AA |
| *cagE* amplification and sequencing | | |
|  | ***cagD*:425U20** | TCA GCA CGA CCA ACA AAC AA |
|  | ***cagF*:112L26** | TCA CCT TCC ATT TCT TCT TCT ATG AA |
|  | *cagE*:424U26 | GCT AAT GAC ATC CAC TTA AAT CCA AA |
|  | *cagE*:1183U25 | AGC GCT ATT GAG ATT AGT GAA TAC G |
|  | *cagE*:2460L23 | CTA AGA CAA ATC TAC GCC CAT CC |
| c*agC* amplification and sequencing | | |
|  | ***cagD*:56L27** | CAT ACG AAC TGA AAA CAA CGA GAC TTA |
|  | **HP0509:1260U24** | CTT GTC TAA AGC CAA ATT CAT GCC |
| *cagA* amplification and sequencing | | |
|  | **J166*cagA* upstream** | CGC TAC CCT TTG TAA TCC TTG |
|  | **J166*cagA* downstream** | GAG AGT TTG GTT CTC ACT CTG TG |
|  | *cagA*:158U26 | ATA AGA ATG ATA GGG ATA ACA GGC AA |
|  | *cagA*:930U25 | GAG TCA TAA TGG CAT AGA ACC TGA A |
|  | *cagA*:1704U21 | AGG ATT GTC CCT ACA AGA AGC |
|  | *cagA*:2554U21 | ACC CTA GTC GGT AAT GGG TTA |
|  | *cagA*:3389U23 | CTG ATT CGT TCA AGT TTT CCA CC |
|  | J99*cagA*:500L24 | GGT TTC CTA TGA TAA TTC CTG CAA |
|  | *cagA*:1209L22 | TTG TGC AAG AAA TTC CAT GAA A |
|  | *cagA*:1937L21 | CTT GAG CTT TTG CTT CCA TTC |
|  | *cagA*:2679L20 | CAT AAA TGG GTT CTC CGC TG |
|  | *cagA*:3329L23 | ATC ATA CTC TGA CAA AGG AAC GC |
